# Chemokine- and chemokine receptor-based signature predicts immunotherapy response in female colorectal adenocarcinoma patients

**DOI:** 10.1038/s41598-023-48623-2

**Published:** 2023-12-04

**Authors:** Wenjie Zhu, Changlei Wu, Shiqi Hu, Sicheng Liu, Shimin Zhao, Dongdong Zhang, Guisheng Qiu, Xiufeng Cheng, Jun Huang

**Affiliations:** 1 Department of Gastrointestinal Surgery, Second Affiliated Hospital of Nanchang University, Nanchang, Jiangxi China; 2 Jiangxi Province Key Laboratory of Molecular Medicine, Second Affiliated Hospital of Nanchang University, Nanchang, Jiangxi China; 3 Department of Critical Care Medicine, First Affiliated Hospital of Nanchang University, Nanchang, Jiangxi China; 4 Queen Mary College, Medical Department, Nanchang University, Nanchang, Jiangxi China

**Keywords:** Cancer, Computational biology and bioinformatics, Immunology, Oncology

## Abstract

The clinical significance and comprehensive characteristics of chemokines and chemokine receptors in female patients with advanced colorectal adenocarcinoma have not ever been reported. Our study explored the expression profiles of chemokines and chemokine receptors and constructed a chemokine- and chemokine receptor-based signature in female patients with advanced colorectal adenocarcinoma. Four independent cohorts containing 1335 patients were enrolled in our study. Univariate Cox regression and least absolute shrinkage and selection operator (LASSO) analyses were performed to construct the signature. CIBERSORT was used to evaluate the landscape of immune cell infiltration. Thirty-two pairs of tissue specimens of female advanced colorectal cancer (CRC) patients and two CRC cell lines were used to validate the signature in vitro. Quantitative real-time PCR and western blotting were performed to validate the mRNA and protein expression levels of signature genes. EdU and colony formation assays were performed to examine proliferative ability. Transwell and wound healing assays were used to evaluate cell invasion and migration capacity. During the signature construction and validation process, we found that the signature was more applicable to female patients with advanced colorectal adenocarcinoma. Hence, the subsequent study mainly focused on the particular subgroup. Enrichment analyses revealed that the signature was closely related to immunity. The landscape of immune cell infiltration presented that the signature was significantly associated with T cells CD8 and neutrophils. Gene set enrichment analysis (GSEA) confirmed that the high-risk group was chiefly enriched in the tumor-promoting related pathways and biological processes, whereas the low-risk group was mainly enriched in anti-tumor immune response pathways and biological processes. The signature was closely correlated with CTLA4, PDL1, PDL2, TMB, MSI, and TIDE, indicating that our signature could serve as a robust biomarker for immunotherapy and chemotherapy response. ROC curves verified that our signature had more robust prognostic power than all immune checkpoints and immunotherapy-related biomarkers. Finally, we used 32 pairs of tissue specimens and 2 CRC cell lines to validate our signature in vitro. We first provided a robust prognostic chemokine- and chemokine receptor-based signature, which could serve as a novel biomarker for immunotherapy and chemotherapy response to guide individualized treatment for female patients with advanced colorectal adenocarcinoma.

## Introduction

As a consequence of dietary patterns, obesity, and unhealthy lifestyles, colorectal cancer (CRC) has become the leading malignant tumor of the digestive system worldwide. CRC also has the third-highest incidence rate and the second-highest mortality rate worldwide^1^. Meanwhile, 30% of patients have developed advanced CRC and lose the opportunity for surgical treatment^2^. Consequently, treating advanced CRC mainly relies on adjuvant immunotherapy and chemotherapy. With the recent application of bioinformatics methods, studies based on multi-omics gene expression data have provided the prognostic evaluation of adjuvant immunotherapy and chemotherapy for CRC patients^3–5^. However, these studies have not considered the effect of different pathological types on the prognosis of patients. CRC is categorized into adenocarcinoma, mucinous neoplasm, serous neoplasm, and other rare types according to pathological differences^6^. Of these types, more than 90% are colorectal adenocarcinoma^7^. Hence, our study focuses on colorectal adenocarcinoma and does not include other types of CRC.

Immune checkpoint inhibitors (ICIs) are the latest and most cutting-edge strategy for cancer treatment. Nowadays, the primary immune checkpoints that are widely used in clinical treatment include cytotoxic programmed death-ligand 1 (PD-L1), programmed death 1 (PD1), and T-lymphocyte-associated protein 4 (CTLA4)^8–10^. In colorectal adenocarcinoma, ICIs have proven to be promising agents^11^. Although advances in the study of ICIs continue, many patients cannot benefit from these medications. ICIs fail to induce a response in approximately two-thirds of patients with multiple types of carcinoma^12^, implying that multiple costimulatory signaling pathways help carcinoma cells escape from immunotherapy in the tumor microenvironment (TME). Consequently, we must explore the latent molecular mechanism and possible biological processes leading to the immune escape of tumor cells in TME.

Chemokines are a large family of small secreted proteins that signal through G-protein-coupled heptahelical chemokine receptors on the cell surface^13^. These receptors are subdivided into four subfamilies (CC, CXC, CX3C, and XC)^14^. Chemokines and their receptors play vital roles in anti-tumor or pro-tumor by affecting TME in CRC. For instance, C-C chemokine receptor 5 (CCR5) blockade can induce macrophage repolarization, triggering anti-tumoral effects in CRC^15^. Macrophages-derived C-C motif chemokine ligand 5 (CCL5) can inhibit the T-cell-mediated killing of CRC and promote the immune escape of CRC^16^. Blockade of C-X-C chemokine receptor 4 (CXCR4) can abrogate the recruitment of innate immune cells to inhibit the development of CRC^17^. Furthermore, chemokines and chemokine receptors influence the effectiveness of ICI therapy. For instance, C-X-C motif chemokine ligand 3 (CXCL3) can bind to C-X-C chemokine receptor 2 (CXCR2) on myeloid-derived suppressor cells and promote their migration to TME, enhance the effectiveness of ICI therapy in CRC^18^. C-X-C motif chemokine ligand 5 (CXCL5) can bind to CXCR2 on cancer-associated fibroblasts (CAFs), thereby promoting the expression of PD-L1 and enhancing the therapeutic effectiveness of anti-PDL1 in CRC^19^. CCL5 deficiency can up-regulate PD-1 and PD-L1 expression and improve the effectiveness of anti-PD-1 and anti-PD-L1 in CRC^20^. Thus, chemokines and chemokine receptors play indispensable roles in TME and immunotherapy of CRC. Consequently, we determined to study the relationships between chemokines and chemokine receptors and TME and immunotherapy in colorectal adenocarcinoma.

No relevant research has been published on the prognostic risk model for chemokines and chemokine receptors in colorectal adenocarcinoma. Therefore, we first establish and validate a chemokine- and chemokine receptor-based prognostic risk model using 1335 cases of colorectal adenocarcinoma from four cohorts. We then explored the relationships between the risk model and TME immune cell infiltration in colorectal adenocarcinoma. To examine the role of our risk model in immunotherapy and chemotherapy, we analyzed the correlations between the risk model and ICIs, tumor mutation burden (TMB), tumor immune dysfunction and exclusion (TIDE) score, and microsatellite instability (MSI). During our study, we inadvertently found that our risk model was more applicable to female patients with advanced colorectal adenocarcinoma. Hence, our study constructs a unique chemokines- and chemokine receptor-based prognostic risk model in female advanced colorectal adenocarcinoma patients, which may be useful to optimize immunotherapies and chemotherapy for the particular population.

## Materials and methods

### Data of colorectal adenocarcinoma patients

The present study included 1135 patients with colorectal adenocarcinoma from four cohorts. Among them, 541 samples with expression data and clinical characteristics were downloaded from the Cancer Genome Atlas (TCGA) (https://portal.gdc.cancer.gov/). These samples from the TCGA cohort were randomly classified into the training cohort (271 samples) and the internal testing cohort (270 samples) at a ratio of 1:1. The other three external independent validation cohorts were collected from Gene Expression Omnibus (GEO) (http://www.ncbi.nlm.nih.gov/geo). These cohorts comprised 177 samples from GSE17536, 55 from GSE17537, and 562 from GSE39582. We also combined four cohorts into an entire cohort (1135 samples), and the batch effects from non-biological technical biases were corrected using the “ComBat” algorithm of the “sva” package. In addition, tissue specimens from 32 CRC patients were collected from the Second Affiliated Hospital of Nanchang University. Our study was approved by the Medical Ethics Committee of the Second Affiliated Hospital of Nanchang University. Informed consent was obtained from all patients enrolled in this trial, and the study met the criteria laid down in the Declaration of Helsinki. The basic clinical characteristics of these cohorts are presented in Table 1.

**Table 1:** Clinical characteristics of colorectal adenocarcinoma from multiple cohorts.

Characteristics	Training cohort (N = 271)	Internal testing cohort (N = 270)	TCGA cohort (N = 541)	GSE17536 cohort (N = 177)	GSE17537 cohort (N = 55)	GSE39582 cohort (N = 562)	Entire cohort (N = 1335)	Tissue specimens (N = 32)
Age								
< = 65	110 (40.59%)	119 (44.07%)	229 (42.33%)	83 (46.89%)	33 (60.00%)	222 (39.50%)	567 (42.47%)	17 (53.13%)
> 65	152 (56.09%)	145 (53.71%)	297 (54.90%)	94 (53.11%)	21 (38.18%)	335 (59.61%)	747 (55.96%)	15 (46.87%)
NA	9 (3.32%)	6 (2.22%)	15 (2.77%)	0 (0%)	1 (1.82%)	5 (0.89%)	21 (1.57%)	0 (0%)
Gender								
Male	134 (49.45%)	145 (44.07%)	279 (51.57%)	96 (54.24%)	26 (47.27%)	305 (54.27%)	706 (52.89%)	0 (0%)
Female	128 (47.23%)	119 (53.71%)	247 (45.66%)	81 (45.76%)	28 (50.91%)	252 (44.84%)	608 (45.54%)	100 (100%)
NA	9 (3.32%)	6 (2.22%)	15 (2.77%)	0 (0%)	1 (1.82%)	5 (0.89%)	21 (1.57%)	0 (0%)
Stage								
I and II	156 (57.57%)	145 (44.07%)	301 (55.64%)	81 (45.76%)	18 (32.73%)	293 (52.14%)	693 (51.91%)	0 (0%)
III and IV	106 (39.11%)	119 (53.71%)	225 (41.59%)	96 (54.24%)	36 (65.45%)	264 (46.97%)	621 (46.52%)	100 (100%)
NA	9 (3.32%)	6 (2.22%)	15 (2.77%)	0 (0%)	1 (1.82%)	5 (0.89%)	21 (1.57%)	0 (0%)

### Identification and signature establishment of six chemokines and chemokine receptors

Fifty-six chemokines and chemokine receptors were enrolled in our study (Supplementary Table 2). Using the “limma” package, we identified 30 differently expressed genes (DEGs) between 44 pairs of colorectal adenocarcinoma tissues and adjacent normal tissues from the TCGA cohort (adjusted *p-*value < 0.05 and |log_2_FC| > 1). The “heatmap” and “ggplot2” packages were used to draw heatmap and Venn diagrams. Next, univariate Cox regression analysis was used to examine the relationships between the expression of chemokines and chemokine receptors and overall survival (OS) in colorectal adenocarcinoma, and six chemokines and chemokine receptors were identified to be closely associated with the prognosis of colorectal adenocarcinoma (Supplementary Table 3). Finally, using the “glmnet” and “survival” packages, least absolute shrinkage and selection operator (LASSO) Cox regression analysis was performed to construct the risk model: Risk score = expression of CCL19 × 0.0423944505338269 + expression of CCL22 × (− 0.633955835342974) + expression of CCR9 × 1.44478984972079 + expression of CXCR5 × 0.0303545048248172 + expression of XCL1 × 0.335066915256399 + expression of CX3CL1 × 0.0321002017091904. The distribution plots of risk score and survival status were used to explore the correlation between risk score and survival status using the “pheatmap” packages.

### Signature validation

Based on the median value of risk score, colorectal adenocarcinoma patients in the training and other testing cohorts were divided into high-risk and low-risk groups, respectively. Using the “survminer” and “survival” packages, Kaplan–Meier survival analyses were performed to validate differences in OS between the high-risk and low-risk groups. Univariate and multivariate Cox regression analyses were performed to validate the prognostic value of our signature.

### Function and pathway enrichment analyses

Gene ontology (GO) and Kyoto encyclopedia of genes and genomes (KEGG) enrichment Analyses were performed using the “clusterProfiler,” “org.Hs.eg.db,” “enrichplot,” and “ggplot2” packages.

### Analyses of immune cell infiltration

CIBERSORT was used to evaluate the abundance of immune cell infiltration using a versatile deconvolution algorithm^21^. LM22 contained 547 genes that can be used to distinguish 22 human immune cell subtypes, which was downloaded from the CIBERSORT web portal (https://cibersort.stanford.edu/)^22^. Based on the LM22 signature algorithm, we used CIBERSORT to calculate the infiltration abundance of 22 types of immune cells in each colorectal adenocarcinoma sample.

### Gene set enrichment analysis (GSEA)

Gene Set Enrichment Analysis (GSEA) is widely used to assess the distribution trend of genes in predefined gene sets, which had been reported to investigate differences in biological processes between distinct groups^23,24^. Thus, we performed GSEA to explore differences in biological processes between the high-risk and low-risk groups. GSEA software was downloaded from the Broad Institute (http://software.broadinstitute.org/gsea/index.jsp)^25^. In addition, the “grid,” “ggplot2,” “gridExtra,” and “plyr” packages were used to combine multiple GSEA results into a single graph.

### Immune checkpoints and tumor immune dysfunction and exclusion (TIDE) analyses

Our study included six common immune checkpoints (PD-1, PDL-1, PDL-2, CTLA4, LAG3, and TIM3) and explored the correlations between immune checkpoints and risk scores. The TIDE score, initially defined by Jiang and his colleagues^26^, has robust power for predicting the prognosis of cancer patients. We obtained the dysfunction and exclusion scores from the TIDE website (http://tide.dfci.harvard.edu). The “ggplot2,” “ggpubr,” and “ggExtra” packages were used to examine the correlations between risk score, immune checkpoints, and TIDE score.

### Tumor mutation burden (TMB), microsatellite instability (MSI), and lymph node analyses

The mutation data of colorectal adenocarcinoma patients were downloaded from TCGA (https://portal.gdc.cancer.gov/). The data regarding MSI and lymph nodes were downloaded from the Cancer Immunome Atlas (TCIA) (https://tcia.at/home). The “ggplot2,” “ggpubr,” and “ggExtra” packages were used to explore the relationships between risk score, TMB, and lymph nodes. The “plyr,” “ggplot2,” and “ggpubr” packages were used to assess the proportion of high- and low-MSI in the high-risk and low-risk groups.

### Receiver operating characteristic (ROC) analyses

Time-dependent ROC curves for 1-, 3-, -5, and 10-year survivals were used to compare the prognostic power between the signature, immune checkpoints, and other biomarkers. The “survival,” “survminer,” and “timeROC” packages were used for analyses.

### Immunohistochemical result

The protein expression levels of human normal colon tissue and colon cancer tissue were determined using the Human Protein Atlas (HPA, https://www.proteinatlas.org/).

### Cell culture and transfection

SW620, SW480, HCT116, HT29, and DLD1 cell lines and the normal colorectal NCM460 cell line were obtained from the Shanghai Institute of Cell Research, Chinese Academy of Sciences. These cell lines were cultured in Dulbecco’s modified Eagle’s medium (Gibco) containing 10% fetal bovine serum (Gibco) at 37 °C and 5% CO_2_. siRNA duplexes against CCR9 were transfected into CRC cells using Lipofectamine 2000 (Invitrogen). The sequences of siRNA duplex sense were as follows:

- si1: 5′-CCCACTTTATTCTGAGGAATA-3′
- si2: 5′-CCAGAAATCTTATACAGCCAA-3′

### Quantitative real-time PCR (qRT-PCR) and western blotting

We extracted total RNA from cells and tissues using the Trizol method, which was reverse transcribed into cDNA (TaKaRa, RR047A). The cDNA was used for real-time quantitative PCR (qRT-PCR; TAKARA, RR420A). The 2^−ΔΔCt^ method was used for data analysis.

The primer sequences used are listed in Supplementary Table 1. Total protein was extracted from HCT116 and SW480 cells. Western blotting was performed using the following primary antibodies: anti-CCR9 (1:1000, Affinity) and anti-GAPDH (1:1000, Proteintech).

### Cell proliferation assay

The proliferative ability of HCT116 and SW480 cells was assessed using the EdU assay and Colony formation assay. Cells were seeded in 96-well plates at 2 × 10^4^ cells per well for the EdU assay. EDU was diluted to 50 μM with complete medium according to the YF^®^594 Click-iT EDU (UE, Shanghai, China) staining kit instructions. The diluted EDU (100 μL) was added to each well and incubated for 2 h. We then removed EDU. Cells in 96-well plates were fixed with 4% paraformaldehyde and neutralized with 2 mg/mL glycine solution; 3% BSA was used to wash cells; 0.5% Triton X-100 was used as a penetration enhancer. Finally, cells were incubated with Click-iT working solution and 1 × Hoechst 33342 solution for 30 min in the dark.

For the colony formation assay, cells were seeded in 6-well plates at 1 × 10^3^ cells per well. The colonies were fixed with 4% paraformaldehyde, stained with crystal violet, and photographed under a microscope.

### Transwell assay and wound healing assay

Transwell invasion and migration assays were used to evaluate cell invasion and migration capacity, respectively. For the Transwell invasion assay, the chambers were coated with Matrigel (1:8 ratio in medium) in advance. For both assays, 2 × 10^4^ cells in 200 μL of serum-free medium were seeded into each upper chamber, whereas 600 µL of complete medium with 10% fetal bovine serum was filled into each lower chamber. After cells were cultured for 24–72 h, the cells in the lower chambers were fixed with 4% paraformaldehyde, stained with crystal violet, and photographed under a microscope.

The wound healing assay is used to evaluate cell migration capacity. Approximately 6 × 10^4^ cells per well were seeded into a 6-well plate. When the cell monolayers were adherent, scratch tests were performed using a 200-μL sterile pipette. The cells were then washed thrice with PBS, added to a serum-free medium, and incubated in an incubator. The scratches were photographed with a microscope at 0 and 24 h.

### Statistical analyses

R studio (version: 4.2.1) and GraphPad Prism (version: 8.0.1) were used for the data analyses. The data were considered to be significant when *p* < 0.05.

### Ethics approval and consent to participate

Our study was approved by the Medical Ethics Committee of the Second Affiliated Hospital of Nanchang University. Informed consent was obtained from all patients enrolled in this trial, and the study met the criteria laid down in the Declaration of Helsinki.

## Results

### Establishment of chemokine- and chemokine receptor-based signature in the training cohort

**Figure 1:**
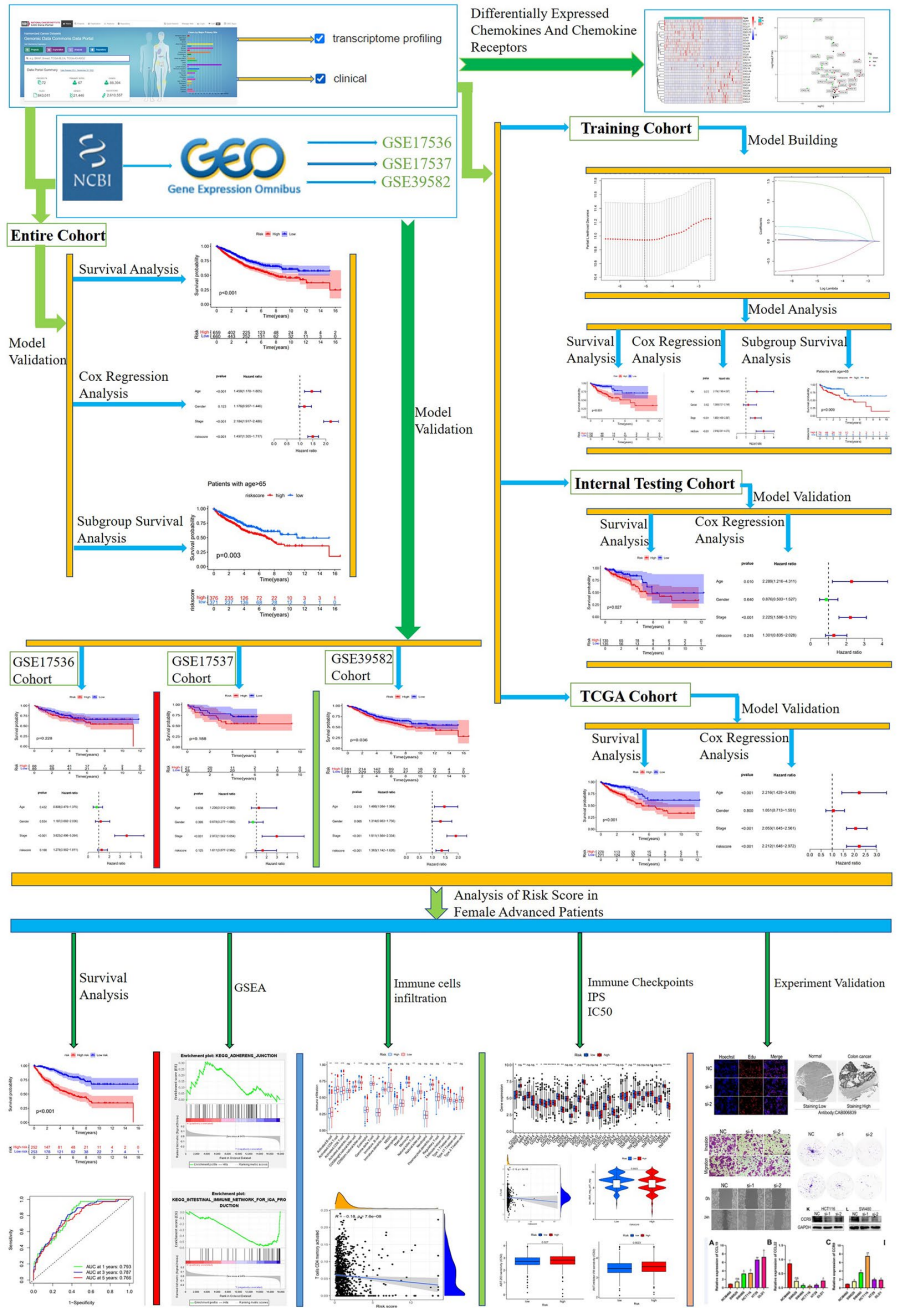
Flow chart of this study.

**Figure 2:**
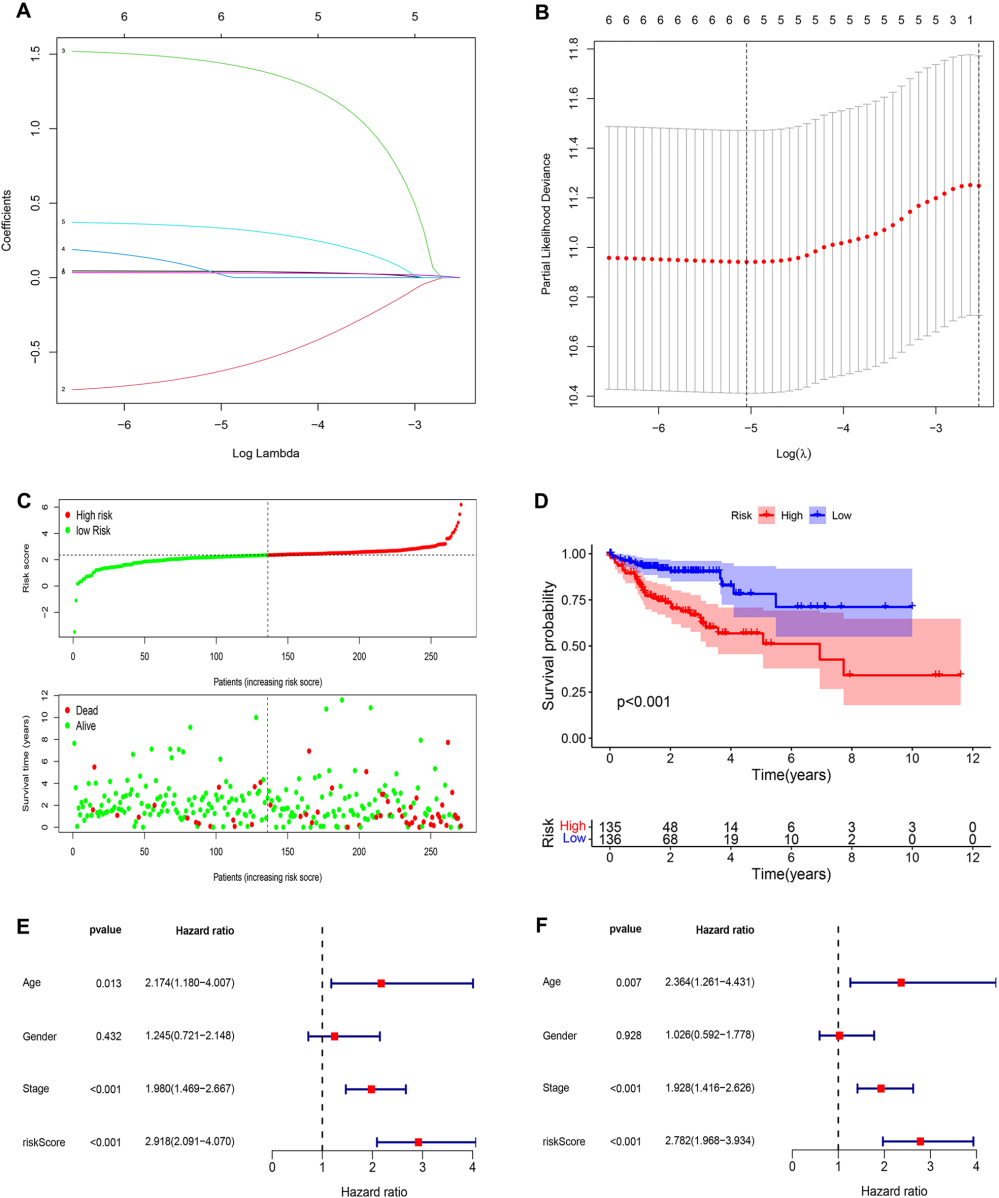
Construction of the chemokines- and chemokine receptors-based signature in training cohort. (**A**) LASSO coefficient profiles of the prognostic genes. (**B**) 100-fold cross-validation for tuning parameter selection in the LASSO model. (**C**) The distribution of risk score and survival status. (**D**) Kaplan–Meier survival analysis compared the OS between colorectal adenocarcinoma patients in high-risk group and colorectal adenocarcinoma patients in low-risk group. (**E**) Univariable Cox regression analysis of risk score, age, gender, and TNM stage. (**F**) Multivariable Cox regression analysis of risk score, age, gender, and TNM stage.

The general process of our study is displayed in Fig. 1. In total, 56 chemokines and chemokine receptors were included in our study (Supplementary Table 2). Heatmap and volcano plot presented the DEGs between 44 pairs of colorectal adenocarcinoma tissues and adjacent normal tissues from the TCGA cohort (Supplementary Fig. S1). A total of 30 DEGs were identified by the threshold of adjusted *p* value < 0.05 and |log_2_FC| > 1. Subsequently, univariate Cox regression analysis was used to explore the relationship between chemokines and chemokine receptors and prognosis in colorectal adenocarcinoma, and six chemokines and chemokine receptors (CCL19, CCL22, CCR9, CXCR5, XCL1, and CX3CL1) were identified as the final predictors for colorectal adenocarcinoma patients’ prognosis (Supplementary Table 3). Finally, based on the six predictors, we performed LASSO Cox regression analysis to construct the risk model (Fig. 2A,B). To make the risk model as simple and reproducible as possible, we used the expression of six predictors to calculate the model’s risk score. The algorithm was as follows: Risk score = expression of CCL19 × 0.0423944505338269 + expression of CCL22 × (− 0.633955835342974) + expression of CCR9 × 1.44478984972079 + expression of CXCR5 × 0.0303545048248172 + expression of XCL1 × 0.335066915256399 + expression of CX3CL1 × 0.0321002017091904.

### Landscape and prognostic significance of the chemokine- and chemokine receptor-based signature in the training cohort

Based on the median risk score, patients with colorectal adenocarcinoma were classified into high-risk and low-risk groups in the training cohort. First, the distribution plots of risk score and survival status are presented in Fig. 2C, indicating more deaths in the high-risk group. To examine the prognostic significance of our signature, we performed Kaplan–Meier survival and Cox regression analyses. The Kaplan–Meier curves showed that the OS of patients with colorectal adenocarcinoma in the high-risk group was observably lower than in the low-risk group (Fig. 2D, p < 0.001). Meanwhile, univariate and multiple Cox regression analyses indicated that risk score was an independent adverse prognostic predictor (Fig. 2E,F, p < 0.001, HR > 1). Therefore, these results prove that a higher risk score is positively related toa worse prognosis in patients with colorectal adenocarcinoma.

### Validation of the chemokine- and chemokine receptor-based signature in testing cohorts

**Figure 3:**
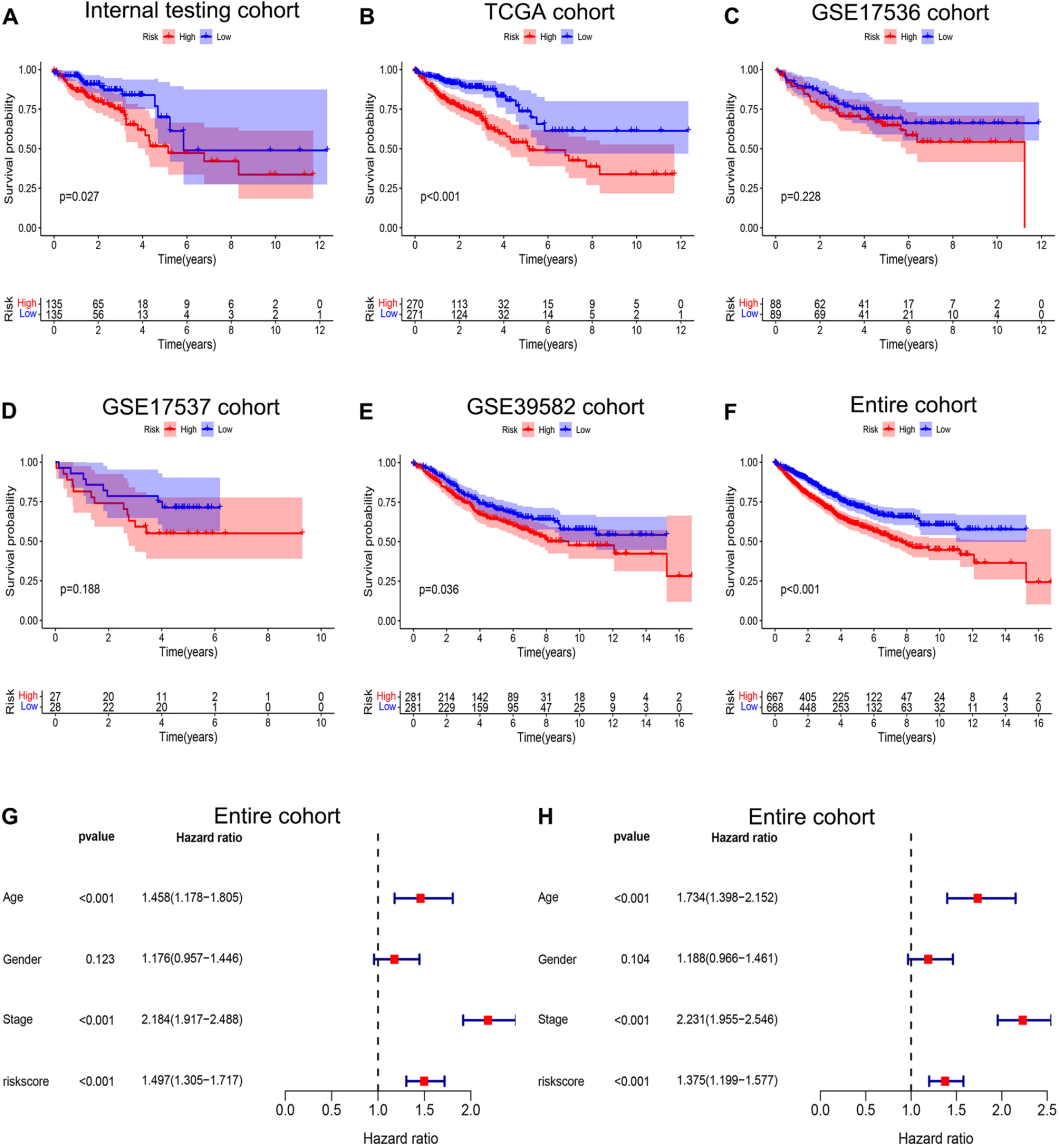
Validation of the chemokines- and chemokine receptors-based signature in internal testing cohort and external testing cohorts. Kaplan–Meier survival analyses compared the OS of colorectal adenocarcinoma patients between high- and low-risk groups in internal testing cohort (**A**), TCGA cohort (**B**), GSE17536 (**C**), GSE17537 (**D**), GSE39582 (**E**), and entire cohort (**F**). (**G**) Univariable Cox regression analysis of risk score, age, gender, and TNM stage in entire cohort. (**H**) Multivariable Cox regression analysis of risk score, age, gender, and TNM stage in entire cohort.

To further validate the prognostic significance of the chemokine- and chemokine receptor-based signature, we performed Kaplan–Meier survival and Cox regression analyses in the following cohorts: internal testing, TCGA, GSE17536, GSE17537, and GSE39582. Kaplan–Meier curves showed that colorectal adenocarcinoma patients in the high-risk group had a shorter OS than those in the low-risk group for the internal testing (Fig. 3A, p = 0.027), TCGA (Fig. 3B, p < 0.001), and GSE39582 cohorts (Fig. 3E, p = 0.036). There was no statistical difference in the GSE17536 (Fig. 3C, p = 0.228) and GSE17537 cohorts (Fig. 3D, p = 0.188). Meanwhile, univariate and multiple Cox regression analyses demonstrated that risk score was an independent adverse prognostic predictor in the TCGA (Supplementary Fig. S2C,D, *p* < 0.001, HR > 1) and GSE39582 cohorts (Supplementary Fig. S3C,D, *p* < 0.001, HR > 1). There was no statistical difference in the internal testing (Supplementary Fig. S2A,B, *p* > 0.05), GSE17536 (Supplementary Fig. S2E,F, *p* > 0.05), and GSE17537 cohorts (Supplementary Fig. S3A,B, *p* > 0.05). Notably, our chemokine- and chemokine receptor-based signature was not statistically significant in cohorts with smaller sample sizes (i.e., GSE17536 and GSE17537 cohorts), whereas it was statistically significant in cohorts with larger sample sizes (i.e., TCGA and GSE39582 cohorts). This finding indicates that the sample size may influence our final result. Thus, we combined four cohorts (the TCGA, GSE17536, GSE17537, and GSE39582 cohorts) into an entire cohort (1135 samples) and then validated the prognostic significance of the chemokine- and chemokine receptor-based signature in the entire cohort. As we expected, Kaplan–Meier curves revealed that colorectal adenocarcinoma patients in the high-risk group had a shorter OS than those in the low-risk group in the entire cohort (Fig. 3F, p < 0.001), and Cox regression analyses indicated that risk score was an independent adverse prognostic predictor in the entire cohort (Fig. 3G,H, p < 0.001, HR > 1). Consequently, the findings from testing cohorts also validate that a higher risk score is positively related to a worse prognosis in patients with colorectal adenocarcinoma. Furthermore, the results confirmed the applicability of the chemokine- and chemokine receptor-based signature to other cohorts.

### Prognostic significance of the chemokine- and chemokine receptor-based signature in distinct subgroups

**Figure 4:**
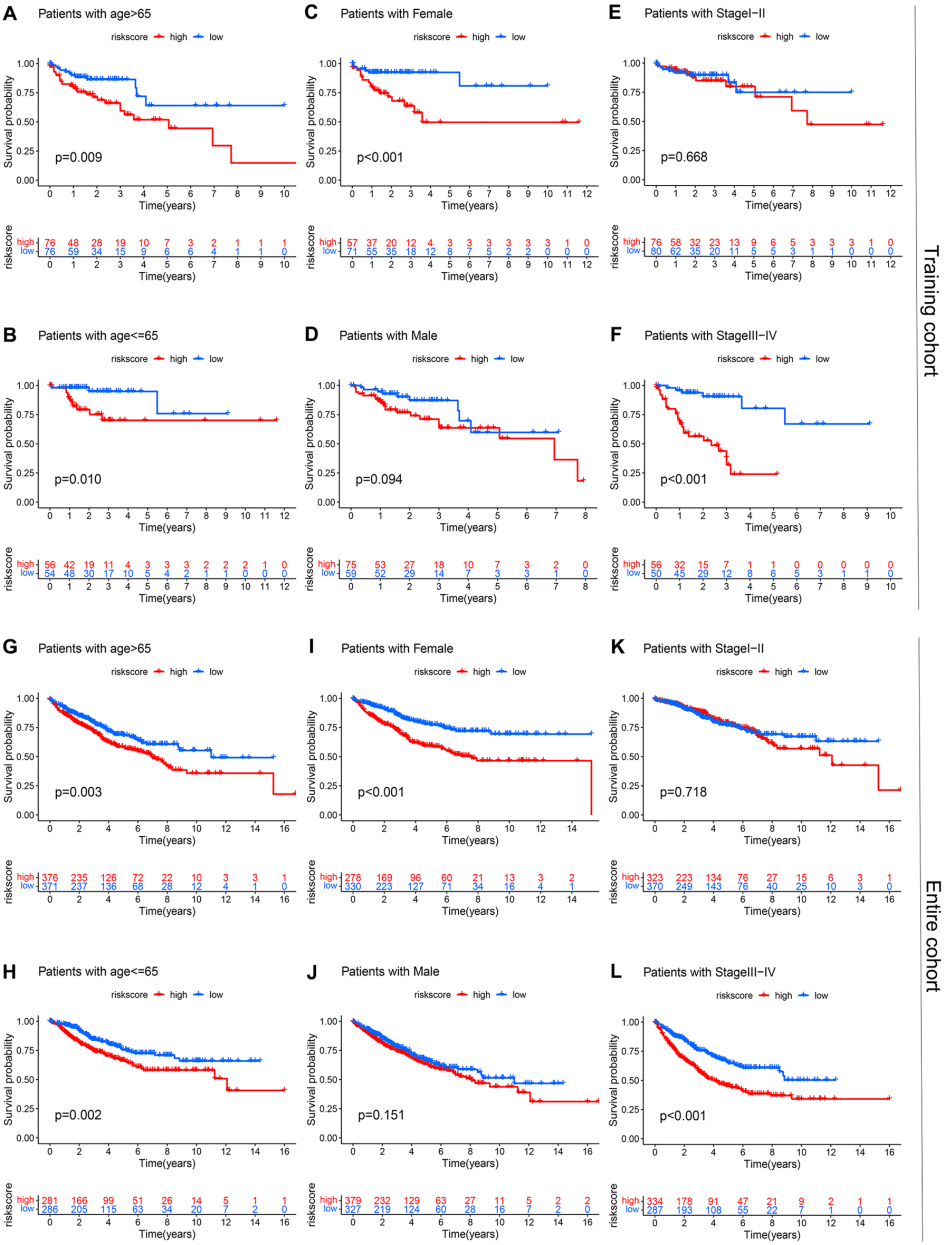
Kaplan–Meier survival analyses compared the OS of colorectal adenocarcinoma patients between high- and low-risk groups in distinct subgroups from training cohort and entire cohort. Kaplan–Meier survival analyses in Age > 65 (**A**), Age < = 65 (**B**), Female (**C**), Male (**D**), Stage I–II (**E**), and Stage III–IV (**F**) subgroups from training cohort. Kaplan–Meier survival analyses in Age > 65 (**G**), Age < = 65 (**H**), Female (**I**), Male (**J**), Stage I–II (**K**), and Stage III–IV (**L**) subgroups from entire cohort.

Studies have found that recurrence and death rates of colorectal carcinoma were higher in females than in males^27^. Meanwhile, the incidence of colorectal carcinoma in the younger population is increasing annually^28^. As known, TNM stage is a significant factor related to prognosis in colorectal carcinoma, colorectal carcinoma patients with stage I–II have a better prognosis than those with stage III–IV^29^. Our study identified age and TMN stage prognostic predictors in colorectal adenocarcinoma (Fig. 3G,H, p < 0.001, HR > 1). Therefore, to exclude the effect of these clinical characteristics on the chemokine- and chemokine receptor-based signature, we further categorized colorectal adenocarcinoma patients in the training cohort into distinct subgroups according to the clinical characteristics and validated the prognostic significance of the chemokine- and chemokine receptor-based signature in these subgroups. The results demonstrated that colorectal adenocarcinoma patients in the high-risk group had a shorter OS than those in the low-risk group for the following subgroups: age > 65 years (Fig. 4A, p = 0.009), age < = 65 years (Fig. 4B, p = 0.01), female (Fig. 4C, p < 0.001), and stage III–IV (Fig. 4F, p < 0.001). However, there was no statistical difference in the male (Fig. 4D, p = 0.094) and stage I–II subgroups (Fig. 4E, p = 0.668). The subgroup sample sizes were relatively small, so the results had no statistical difference in the male and stage I–II subgroups because of the small sample size. To rule out the possibility, we classified colorectal adenocarcinoma patients in the entire cohort into distinct subgroups according to the clinical characteristics and then validated the prognostic significance of the chemokine- and chemokine receptor-based signature in these subgroups. the results also demonstrated that colorectal adenocarcinoma patients in the high-risk group had a shorter OS than those in the low-risk group for the following subgroups: age > 65 years (Fig. 4G, p = 0.003), age < = 65 years (Fig. 4H, p = 0.002), female (Fig. 4I, p < 0.001), and stage III–IV (Fig. 4L, p < 0.001). There was also no statistical difference in the male (Fig. 4J, p = 0.151) and stage I–II subgroups (Fig. 4K, p = 0.718). Consequently, the non-significance of our results in the male and stage I–II subgroups was independent of the sample size, indicating that our risk model is more applicable to female patients with advanced (stage III–IV) colorectal adenocarcinoma.

### Prognostic significance of the chemokine- and chemokine receptor-based signature in the female + stage III–IV cohort

**Figure 5:**
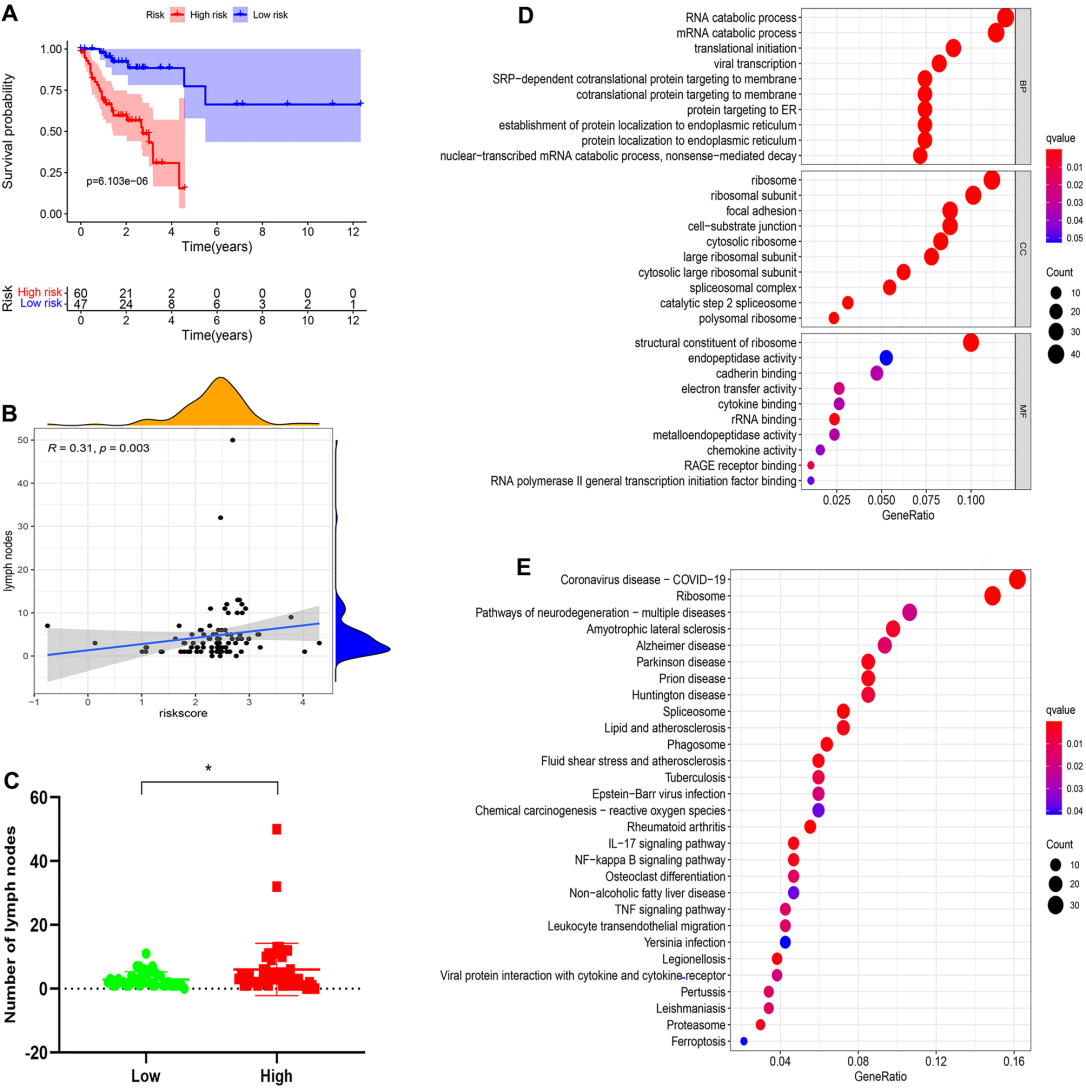
Kaplan–Meier survival, lymph nodes, and enrichment analyses in Female + Stage III/IV patients from TCGA. (**A**) Kaplan–Meier survival analyses compared the OS of female advanced colorectal adenocarcinoma patients between high- and low-risk groups. (**B**) Correlation between risk score and number of metastatic lymph nodes. (**C**) Difference in the number of metastatic lymph nodes between high- and low-risk groups. 398 DEGs identified between high- and low-risk groups were used to perform GO (**D**) and KEGG (**E**) enrichment analyses.

Subsequently, to validate that our risk model was more applicable to female advanced colorectal adenocarcinoma patients, we extracted 107 female advanced colorectal adenocarcinoma patient data from TCGA and named it the Female + Stage III–IV cohort. In the Female + Stage III–IV cohort, Kaplan–Meier survival analysis demonstrated that the OS of female patients with advanced colorectal adenocarcinoma in the high-risk group was significantly shorter than those in the low-risk group (Fig. 5A, p = 6.103e−06). Furthermore, we explored the correlation between risk score and metastatic lymph nodes in the Female + Stage III–IV cohort. The number of metastatic lymph nodes was negatively correlated with the prognosis of colorectal carcinoma patients^30^. In our study, the risk score was positively correlated with the number of metastatic lymph nodes in colorectal adenocarcinoma (Fig. 5B, p = 0.003, R = 0.31). Meanwhile, the number of metastatic lymph nodes in the high-risk group was significantly more abundant than those in the low-risk group (Fig. 5C, p < 0.05). Consequently, these findings suggest that a higher risk score is positively associated with poorer prognosis among female patients with advanced colorectal adenocarcinoma.

### GO and KEGG enrichment analyses

We wondered which latent mechanisms were responsible for the prognostic difference between the high-risk and low-risk groups. To explore these latent mechanisms, we identified 398 DEGs between the high-risk and low-risk groups (Supplementary Table 4, adjusted *p*-value < 0.05 and |log_2_FC| > 1). GO and KEGG enrichment analyses were performed on these 398 DEGs. GO enrichment analysis revealed these DEGs were closely related to cytokine binding and chemokine activity (Fig. 5D). Meanwhile, KEGG enrichment analyses revealed that these DEGs were mainly enriched in immune activation-related pathways (i.e., IL-17 signaling pathway, NF-kappa B signaling pathway, TNF signaling pathway, and leukocyte transendothelial migration) (Fig. 5E). Hence, these findings suggest that the prognostic difference between the high- and low-risk groups is closely related to immunity.

### The immune cell infiltration profile of the chemokine- and chemokine receptor-based signature in the female + stage III–IV cohort

**Figure 6:**
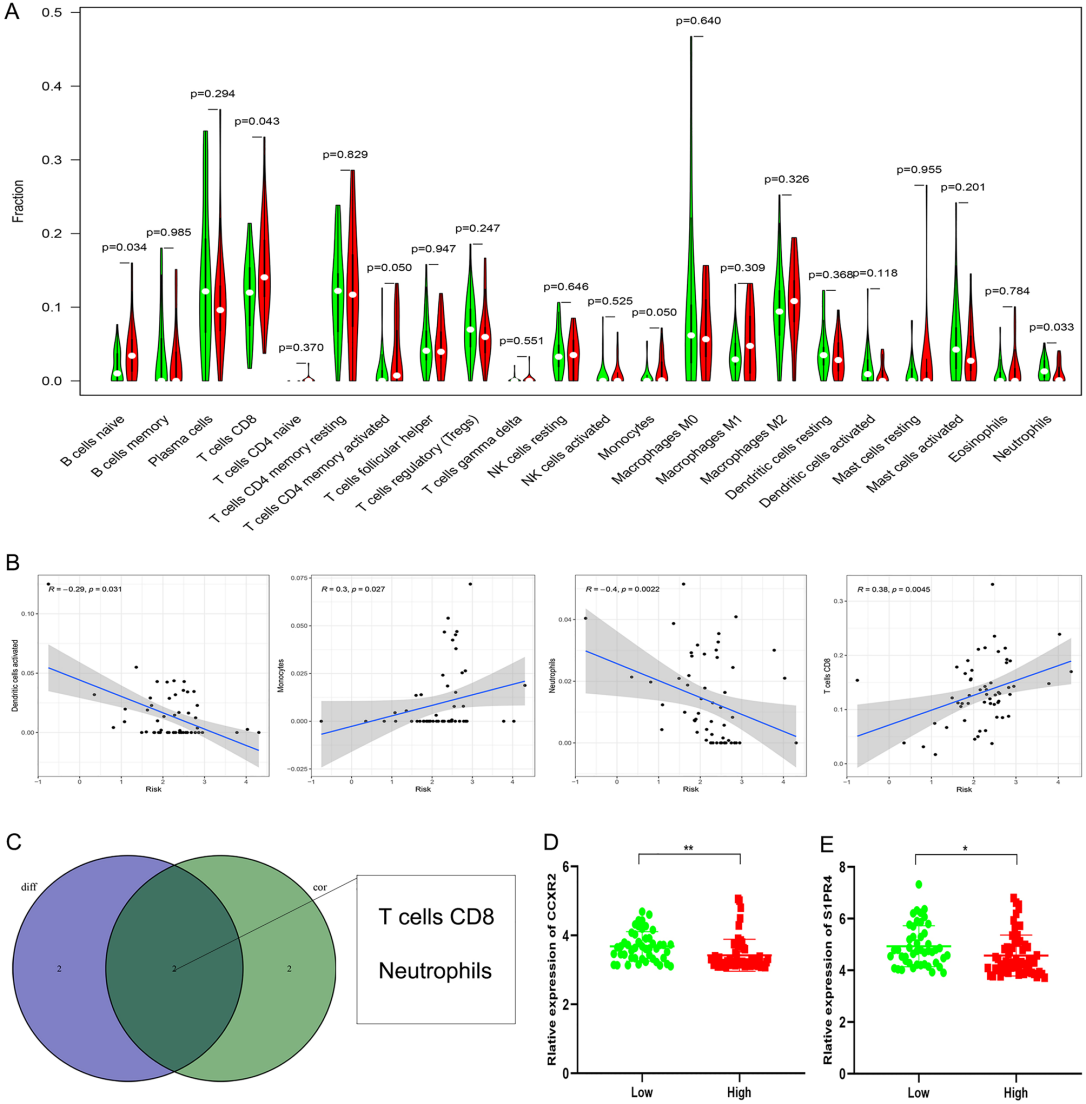
The immune cell infiltration landscape of the chemokines- and chemokine receptors-based signature in Female + Stage III/IV patients from TCGA. (**A**) The infiltration differences of 22 immune cells between high- and low-risk groups. (**B**) Correlations between risk score and the infiltration level of estimated immune cells. (**C**) Venn diagram showed the significant immune cells in both difference and correlation analyses. (**D**) The differential expression of CCXR2 between high- and low-risk groups. (**E**) The differential expression of S1PR4 between high- and low-risk groups. *p < 0.05; **p < 0.01; ***p < 0.001.

To confirm that the prognostic difference between the high-risk and low-risk groups was closely related to immunity, we analyzed the infiltration of immune cells between the high-risk and low-risk groups in the Female + Stage III–IV cohort. The percentage of immune cell infiltration in each TCGA sample is shown in Supplementary Fig. S4. We also verified the correlation between risk score and immune cell infiltration in the Female + Stage III–IV cohort. As shown in Fig. 6A, compared with female patients with advanced colorectal adenocarcinoma in the high-risk group, those in the low-risk group had a higher proportion of neutrophils. However, B cells naive, T cells CD8, and T cells CD4 memory activated had a higher proportion in the high-risk group. Meanwhile, dendritic cells (DCs) activated and neutrophils were negatively related to risk score, whereas monocytes and T cells CD8 were positively correlated with risk score (Fig. 6B). Subsequently, we intersected statistically significant results in Fig. 6A,B. Venn diagram showed that only T cells CD8 and neutrophils had statistical significance in the two results (Fig. 6C). Generally, a higher infiltration of T cells CD8 was positively correlated with better prognosis in colorectal carcinoma patients^31^. To our confusion, T cells CD8 was positively related to risk score in our study, which was contrary to our expected result. Therefore, we suspected other potential factors might influence the infiltration of T cells CD8 in colorectal carcinoma. From previous studies, we identified CCXR2 and S1PR4 as the factors affecting the infiltration of T cells CD8 in colorectal carcinoma. Overexpression of CCXR2 could inhibit infiltration of T cells CD8 in colorectal carcinoma^32^. Similarly, S1PR4 knockdown could enhance infiltration of T cells CD8^33^. Consequently, we speculated that the low-risk group could reverse the T cells CD8 infiltration by up-regulating the expression of CCXR2 and S1PR4, resulting in a lower infiltration level of T cells CD8 in the low-risk group. To test our speculation, we verified the expression of CCXR2 and S1PR4 in the high-risk and low-risk groups, respectively. Consistent with our assumption, the expression of CCXR2 was significantly higher in the low-risk group than in the high-risk group (Fig. 6D, p < 0.01). Meanwhile, S1PR4 expression was significantly higher in the low-risk group than in the high-risk group (Fig. 6E, p < 0.05). Thus, our results demonstrate that the low-risk group could reverse the T cells CD8 infiltration by up-regulating the expression of CCXR2 and S1PR4 in female patients with advanced colorectal adenocarcinoma. Undoubtedly, there exist many additional costimulatory factors that warrant further investigation.

### GSEA analyses of the chemokine- and chemokine receptor-based signature in the female + stage III–IV cohort

**Figure 7:**
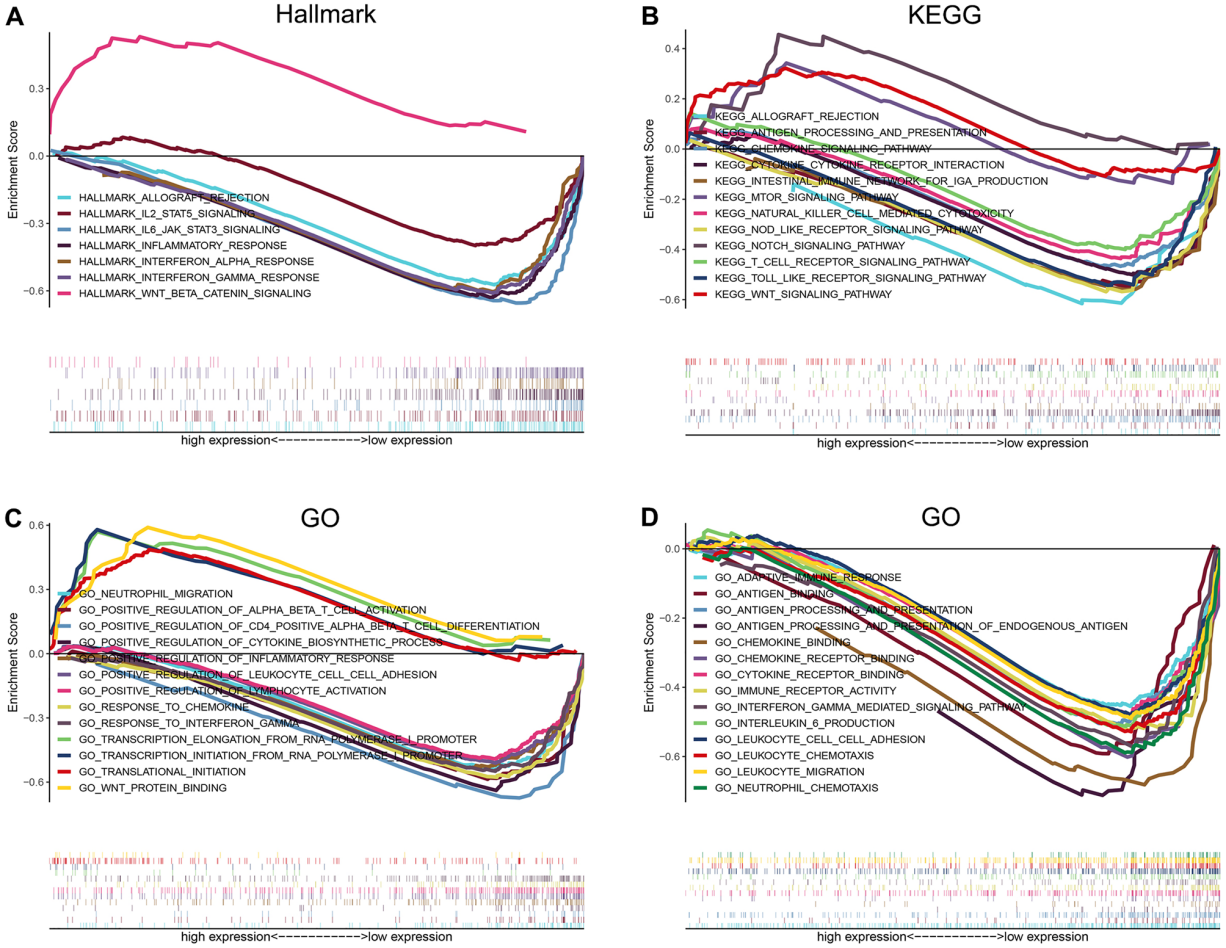
GSEA of high- and low-risk groups based on Hallmark (**A**), KEGG (**B**), and GO (**C**,**D**) gene sets in Female + Stage III/IV patients from TCGA.

Although we previously performed GO and KEGG enrichment analyses to demonstrate that our risk model was closely associated with immunity, and the analysis of immune cell infiltration further confirmed that our model was prominently related to infiltration of T cells CD8 and neutrophils, the immune-related pathways and biological processes in distinct risk groups were not clear. We performed GSEA analyses to explore the corresponding pathways and biological processes in the high-risk and low-risk groups. As shown in Fig. 7A, GSEA analysis based on hallmark gene sets suggested that the tumor-promoting pathways (i.e., WNT_BETA_CATENIN_SIGNALING) were enriched in the high-risk group, whereas abundant anti-tumor immune response-related pathways (i.e., ALLOGRAFT_REJECTION, IL2_STAT5_SIGNALING, and IL6_JAK_STAT3_SIGNALING) and inflammatory response-related pathway (i.e., INFLAMMATORY_RESPONSE, INTERFERON_GAMMA_RESPONSE, and INTERFERON_ALPHA_RESPONSE) were enriched in the low-risk group. Meanwhile, the GSEA analysis based on KEGG gene sets demonstrated that the tumor-promoting pathways (i.e., NOTCH_SIGNALING_PATHWAY, WNT_SIGNALING_PATHWAY, and MTOR_SIGNALING_PATHWAY) were enriched in the high-risk group, whereas abundant anti-tumor immune response-related pathways (i.e., T_CELL_RECEPTOR_SIGNALING_PATHWAY, NATURAL_KILLER_CELL_MEDIATED_CYTOTOXICITY, INTESTINAL_IMMUNE_NETWORK_FOR_IGA_PRODUCTION, ANTIGEN_PROCESSING_AND_PRESENTATION, NOD_LIKE_RECEPTOR_SIGNALING, and ALLOGRAFT_REJECTION) were enriched in the low-risk group (Fig. 7B). Similarly, the GSEA analysis based on GO gene sets disclosed that the tumor-promoting related biological processes (WNT_PROTEIN_BINDING) and transcription-related biological processes (i.e., TRANSLATIONAL_INITIATION, TRANSCRIPTION_INITIATION_FROM_RNA_POLYMERASE_I_PROMOTER, and TRANSCRIPTION_ELONGATION_FROM_RNA_POLYMERASE_I_PROMOTER) were enriched in the high-risk group, whereas abundant anti-tumor immune response-related biological processes (i.e., POSITIVE_REGULATION_OF_LYMPHOCYTE_ACTIVATION, POSITIVE_REGULATION_OF_CD4_POSITIVE_ALPHA_BETA_T_CELL_DIFFERENTIATION, POSITIVE_REGULATION_OF_ALPHA_BETA_T_CELL_ACTIVATION, NEUTROPHIL_MIGRATION, ADAPTIVE_IMMUNE_RESPONSE, ANTIGEN_PROCESSING_AND_PRESENTATION, IMMUNE_RECEPTOR_ACTIVITY, and NEUTROPHIL_CHEMOTAXIS) and inflammatory response-related pathway (i.e., GO_RESPONSE_TO_INTERFERON_GAMMA, POSITIVE_REGULATION_OF_INFLAMMATORY_RESPONSE, and INTERFERON_GAMMA_MEDIATED_SIGNALING_PATHWAY) were enriched in the low-risk group (Fig. 7C,D). The enrichment score (NES) and nominal *p*-value are presented in Supplementary Table 5. Consequently, these results of GSEA analyses confirmed why the prognosis of colorectal adenocarcinoma patients in the low-risk group was better than that in the high-risk group from the perspective of molecular biology.

### Relationships between the chemokine- and chemokine receptor-based signature and immune checkpoints in the female + stage III–IV and TCGA cohorts

**Figure 8:**
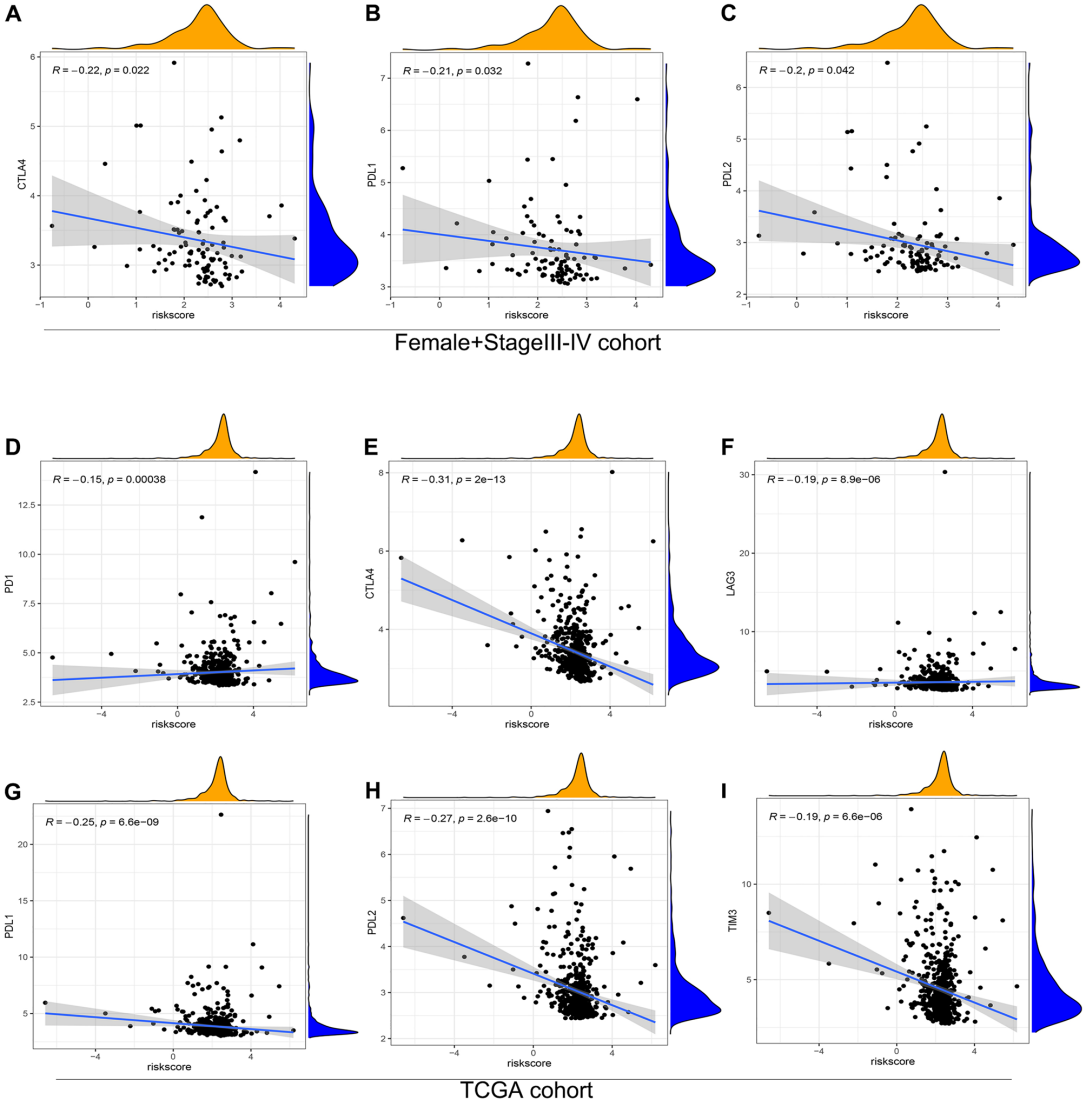
Correlations between risk score and immune checkpoints in Female + Stage III/IV patients from TCGA and all patients from TCGA. Correlations between risk score and CTLA4 (**A**), PDL1 (**B**), and PDL2 (**C**) in Female + Stage III/IV patients from TCGA. Correlations between risk score and PD1 (**D**), CTLA4 (**E**), LAG3 (**F**), PDL1 (**G**), PDL2 (**H**), and TIM3 (**I**) in all patients from TCGA.

According to the aforementioned results, we proved that our risk model was closely associated with immunity. Therefore, we explored whether our risk model could be used as a basis for colorectal adenocarcinoma patients to receive immunotherapy. Immune checkpoint inhibitors (ICIs) have become the first-line treatment for advanced colorectal adenocarcinoma. Major immune checkpoints such as PD1, CTLA4, PDL1, PDL2, LAG3, and TIM3 have been commonly used as biomarkers of immunotherapy response^34,35^. In our study, the risk score was negatively correlated with the expression of CTLA4 (Fig. 8A, p = 0.022, R = − 0.22), PDL1 (Fig. 8B, p = 0.032, R = − 0.21), and PDL2 (Fig. 8C, p = 0.042, R = − 0.2) in the Female + Stage III–IV cohort. However, there was no statistical significance in PD1 (Supplementary Fig. S5A, *p* = 0.44, R = − 0.076), LAG3 (Supplementary Fig. S5B, *p* = 0.052, R = − 0.19), and TIM3 (Supplementary Fig. S5C, *p* = 0.16, R = − 0.14). Furthermore, we validated our results in the TCGA cohort. Notably, our results demonstrated that risk score was negatively correlated with all six immune checkpoints in the TCGA cohort (Fig. 8D–I, p < 0.042, R < 0). The immunotherapy efficacy of ICIs is positively correlated with the expression of immune checkpoints in tumors^36^. Consequently, our results prove that female advanced colorectal adenocarcinoma patients with lower risk scores are more likely to benefit from immunotherapy of anti-CTLA-4, anti-PDL1, and anti-PDL2 than those with higher risk scores. Meanwhile, colorectal adenocarcinoma patients with lower risk scores are more likely to benefit from immunotherapy of all six ICIs than those with higher risk scores.

### Relationships between the chemokine- and chemokine receptor-based signature and other immunotherapy-related biomarkers in the female + stage III–IV cohort

**Figure 9:**
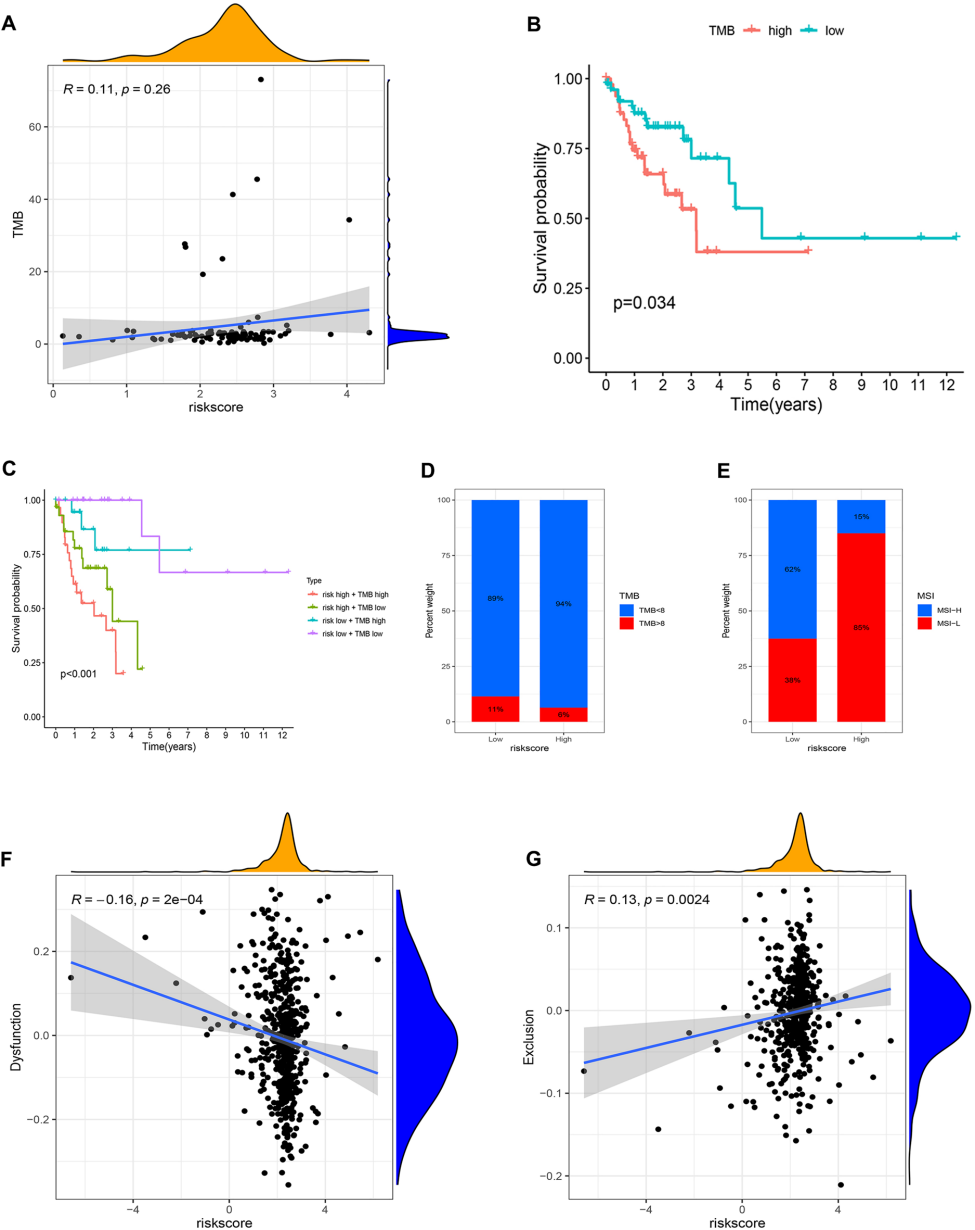
Relationships between the chemokines- and chemokine receptors-based signature and other immunotherapy-related biomarkers in Female + Stage III/IV patients from TCGA. (**A**) Correlation between risk score and TMB. (**B**) Kaplan–Meier survival analysis compared the OS of female advanced colorectal adenocarcinoma patients between high and low TMB groups. (**C**) Kaplan–Meier survival analysis compared the OS of female advanced colorectal adenocarcinoma patients among high-risk + high TMB, high risk + low TMB, low risk + high TMB, and low risk + low TMB. (**D**) The differential proportions of TMB < 8 and TMB > 8 between high- and low-risk groups. (**E**) The differential proportions of high and low MSI between high- and low-risk groups. (**F**) Correlation between risk score and T cell dysfunction score. (**G**) Correlation between risk score and T cell exclusion score.

To further explore whether our risk model could be used as a basis for female advanced colorectal adenocarcinoma patients to receive immunotherapy, we investigated relationships between the chemokine- and chemokine receptor-based signature and other immunotherapy-related biomarkers (TMB, MSI, and TIDE) in the Female + Stage III–IV cohort. A previous study found that colorectal carcinoma patients with higher TMB have better prognoses and better response to immunotherapy than those with lower TMB^37^. However, in our study, there was no significant correlation between risk score and TMB (Fig. 9A, p = 0.26, R = 0.11). Notably, the OS of colorectal adenocarcinoma patients in the high-TMB group was lower than those in the low-TMB group (Fig. 9B, p = 0.034), contrary to our expected result. Only a few studies have reported the correlation between TMB and the prognosis of colorectal carcinoma patients. Some researchers have demonstrated that higher TMB is closely related to worse prognosis in prostate cancer^38^ and clear cell renal cell carcinoma^39^. Consequently, the effect of TMB on the prognosis of tumor patients is still controversial. In other words, future research with a larger sample size will be required to accurately elucidate the relationship between TMB and the prognosis of patients with colorectal adenocarcinoma. Subsequently, we classified patients in the high- and low-TMB groups into four distinct subgroups according to the risk score, and we performed Kaplan–Meier survival analyses on the four subgroups. The results demonstrated that female patients with advanced colorectal adenocarcinoma in the low-risk and low-TMB groups had the best prognosis, whereas those in the high-risk and high-TMB groups had the worst prognosis (Fig. 9C, p < 0.001). Meanwhile, the OS of female patients with advanced colorectal adenocarcinoma in the low-risk and high-TMB groups was significantly longer than that in the high-risk and high-TMB groups (Fig. 9C, p < 0.001), indicating that a lower risk score could reverse the worse prognosis caused by high TMB. A study verified that adjuvant chemotherapy for colorectal carcinoma patients with TMB > 8 can prominently improve their survival time^40^. Therefore, we examined the proportion of colorectal adenocarcinoma patients with TMB > 8 and TMB < 8 in the high-risk and low-risk groups and found that the proportion of female advanced colorectal adenocarcinoma patients with TMB > 8 was significantly higher in the low-risk group (11%) than that in the high-risk group (6%) (Fig. 9D). Consequently, the findings indicate that female advanced colorectal adenocarcinoma patients in the low-risk group are more likely to benefit from chemotherapy compared with those in the high-risk group. Indeed, further clinical trials are required to confirm the conclusion.

The level of MSI is positively correlated with the sensitivity of immunotherapy in colorectal carcinoma patients^41^. Meanwhile, the level of MSI is positively related to the efficiency of chemotherapy in colorectal carcinoma patients^42,43^. Therefore, we examined the proportion of colorectal adenocarcinoma patients with high MSI and low MSI in the high-risk and low-risk groups and found that the proportion of female advanced colorectal adenocarcinoma patients with high MSI was prominently higher in the low-risk group (62%) than that in the high-risk group (15%) (Fig. 9E). Consequently, the result indicates that female advanced colorectal adenocarcinoma patients in the low-risk group are more likely to benefit from immunotherapy and chemotherapy compared with those in the high-risk group.

TIDE is a more accurate biomarker than TMB, MSI, and ICIs, containing T-cell dysfunction and exclusion scores^26^. T-cell dysfunction was presented in tumors with high infiltration of cytotoxic T lymphocytes (CTL), whereas T-cell exclusion was in tumors with low T-cell invasion^26^. In other words, a higher T-cell dysfunction score was significantly correlated with more sensitivity of immunotherapy and better prognosis in tumors, whereas a higher T-cell exclusion score was closely associated with worse efficacy of immunotherapy and prognosis in tumors. In our study, risk score was negatively correlated with T-cell dysfunction score (Fig. 9F, p = 2e−04, R = − 0.16), whereas risk score was positively related to T-cell exclusion score (Fig. 9G, p = 0.0024, R = 0.13). Consequently, the results indicate again that colorectal adenocarcinoma patients with higher risk scores are more likely to benefit from immunotherapy compared with patients in the high-risk group.

### Comparison of the prognostic power of the chemokine- and chemokine receptor-based signature with immune checkpoints and other biomarkers in the female + stage III–IV cohort

**Figure 10:**
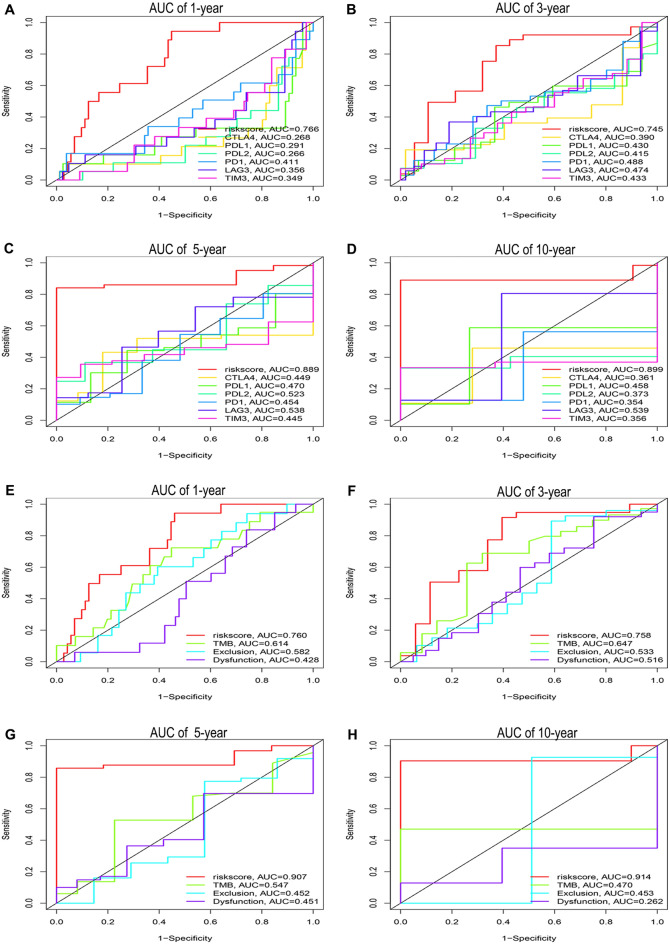
Comparison of the prognostic power of the chemokines- and chemokine Receptors-based signature with immune checkpoints and other biomarkers in Female + Stage III/IV patients from TCGA. AUC of 1 year (**A**), 3 years (**B**), 5 years (**C**), and 10 years (**D**) of risk score, CTLA4, PD1, PDL1, PDL2, LAG3, and TIM3. AUC of 1 year, 3 years (**F**), 5 years (**G**), and 10 years (**H**) of risk score, TMB, T cells exclusion score, and T cells dysfunction score.

To assess the advantage of our risk model in predicting the prognosis of female advanced colorectal adenocarcinoma patients, we compared the risk score with immune checkpoints and other biomarkers through receiver operating characteristic (ROC) analyses in the Female + Stage III–IV cohort. The results demonstrated that the area under the curve (AUC) of 1 year (Fig. 10A, AUC = 0.766), 3 years (Fig. 10B, AUC = 0.745), 5 years (Fig. 10C, AUC = 0.889), and 10 years (Fig. 10D, AUC = 0.899) in risk score was higher than that in all six immune checkpoints. Meanwhile, the AUC of 1 year (Fig. 10E, AUC = 0.760), 3 years (Fig. 10F, AUC = 0.758), 5 years (Fig. 10G, AUC = 0.907), and 10 years (Fig. 10H, AUC = 0.914) in risk score was higher than that in TMB, T-cell dysfunction score, and T-cell exclusion score. Consequently, the results confirm that the prognostic power of our risk model is more robust than that of immune checkpoints and other biomarkers.

### Prediction of chemotherapeutic drug sensitivity, establishment of nomogram, and validation of signature

**Figure 11:**
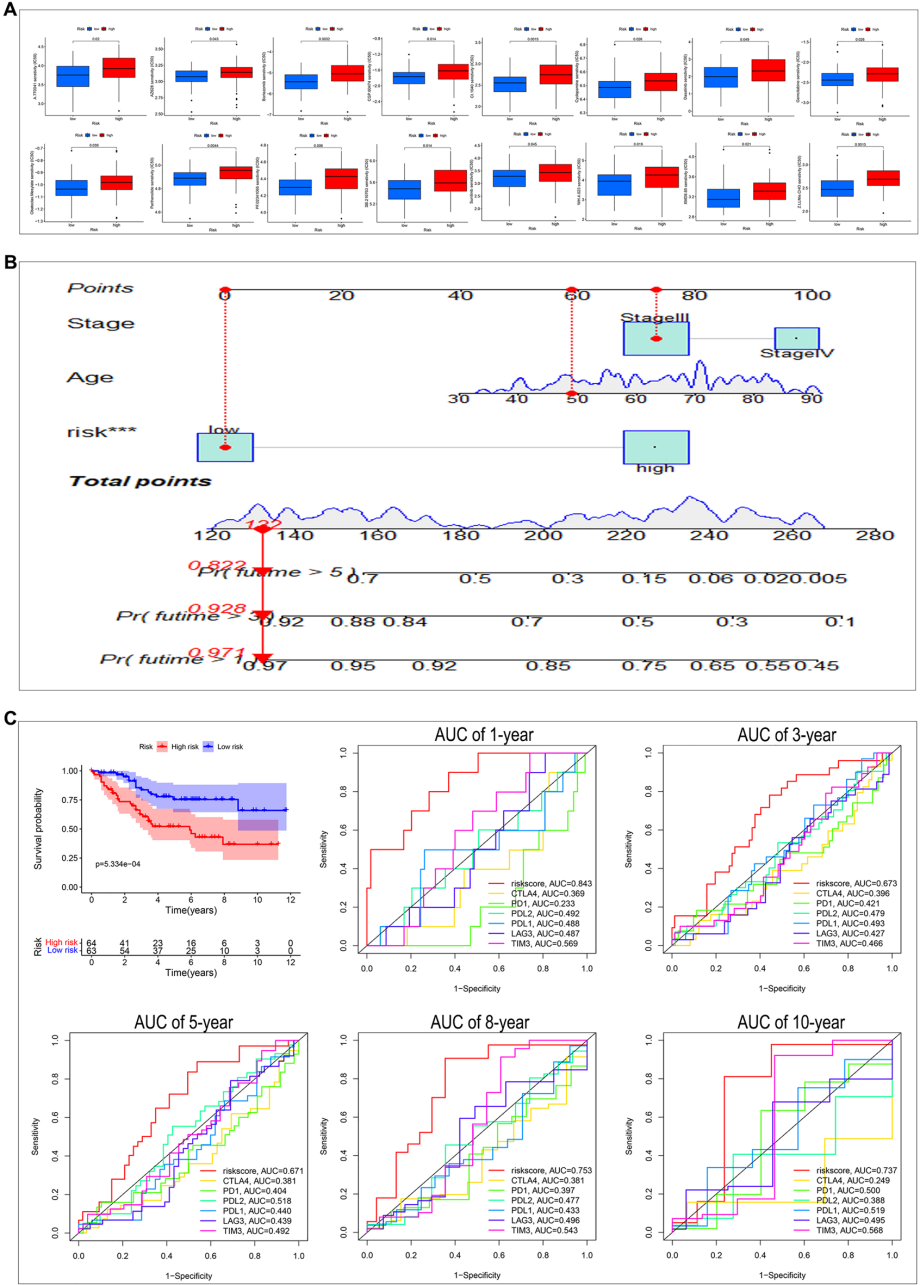
Signature predicts chemotherapy response and nomogram estimates OS of Female + Stage III/IV patients from TCGA and the validation of signature in Female + Stage III/IV patients from GSE39582. (**A**) Difference in IC50 between groups high- and low-risk group for 16 chemotherapeutic agents. (**B**) A nomogram consisting of age, stage, and risk score. (**C**) Validation of signature in Female + Stage III/IV patients from GSE39582.

To explore the difference in sensitivity to chemotherapeutic drugs between the high-risk and low-risk groups, we predicted the IC50 values of multiple chemotherapeutic drugs. As displayed in Fig. 11A, the IC50 values of multiple drugs were lower in the low-risk group than in the high-risk group, suggesting that CRC patients with lower risk scores are more sensitive to treatment of chemotherapeutic drugs than those with higher risk scores.

Subsequently, considering the inconvenient clinical utility of risk score in predicting the prognosis of CRC patients, a nomogram containing risk score, age, and TMN stage was constructed to forecast 1-, 3- and 5-year OS of CRC patients (Fig. 11B). In addition, we also used female advanced CRC patients from GSE39582 to validate the applicability of our signature (Fig. 11C). The K–M curve of OS indicated that the higher the risk score, the lower the OS. And the AUC of 1-, 3-, 5-, 8-, and 10-year in risk score was higher than that in all six immune checkpoints. Hence, these results demonstrated that our signature could be used in other datasets to predict female advanced CRC patients' OS and immunotherapy outcomes.

### Verification of mRNA expression levels of signature genes and adverse effects of CCR9 in CRC cell lines and tissues

**Figure 12:**
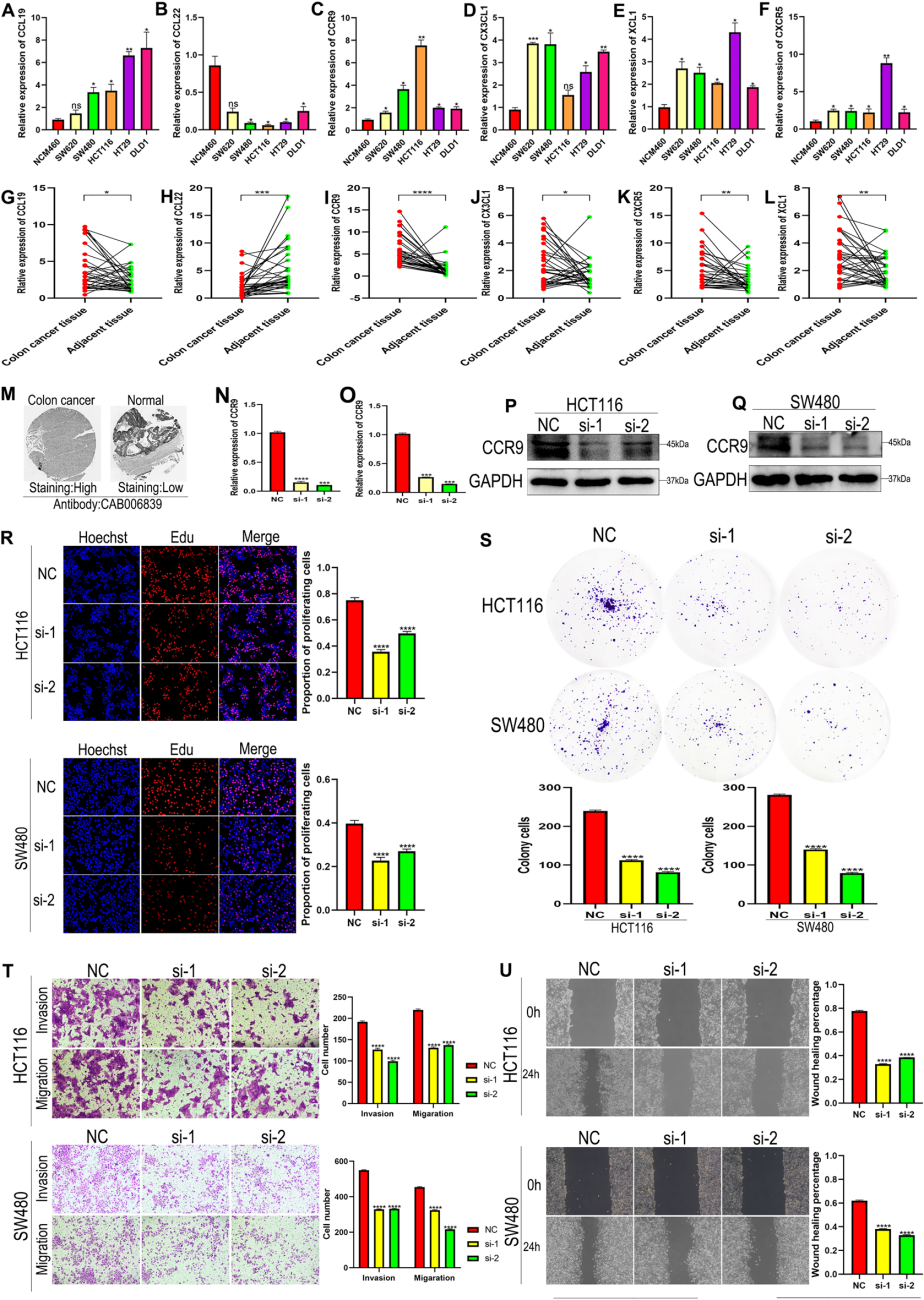
Verify the mRNA expression levels of the six signature genes and adverse effects of CCR9 in CRC cell lines and tissues. The mRNA expression levels of CCL19 (**A**), CCL22 (**B**), CCR9 (**C**), CX3CL1 (**D**), XCL1 (**E**), and CXCR5 (**F**) in CRC cell lines. The mRNA expression levels of CCL19 (**G**), CCL22 (**H**), CCR9 (**I**), CX3CL1 (**J**), XCL1 (**K**), and CXCR5 (**L**) in 36 pairs of female advanced CRC tissues. (**M**) The IHC of CCR9 in normal tissue and colon cancer from HPA database. Verification of the efficiency of CCR9 knockdown using qRT-PCR (**N**) and Western blotting (**P**) in HCT116 cell line. Verification of the efficiency of CCR9 knockdown using qRT-PCR (**O**) and Western blotting (**Q**) in SW480 cell line. EdU staining (**R**) and plate cloning assay (**S**) verified proliferative capacity of CRC cell lines. (**T**) Transwell invasion assay was used to verify the invasion ability of CRC cell lines. (**T**,**U**) Transwell migration assay and scratch healing assay were used to verify the migration ability of CRC cell lines.

We verified the mRNA expression levels of the six signature genes in normal and CRC cell lines using qRT-PCR. The results demonstrated that CCL22 (Fig. 12B) was lowly expressed in CRC cell lines, whereas CCL19 (Fig. 12A), CCR9 (Fig. 12C), CX3CL1 (Fig. 12D), XCL1 (Fig. 12E), and CXCR5 (Fig. 12F) were highly expressed in CRC cell lines. In addition, we collected tissue specimens from 32 female advanced CRC patients at the Second Affiliated Hospital of Nanchang University, and the result of qRT-PCR also showed that the expression level of CCL22 (Fig. 12H) was lower in female advanced CRC tissues than adjacent tissues, whereas the expression level of CCL19 (Fig. 12G), CCR9 (Fig. 12I), CX3CL1 (Fig. 12J), XCL1 (Fig. 12K), and CXCR5 (Fig. 12L) were higher in female advanced CRC tissues than adjacent tissues.

Because of the importance of CCR9 in the six signature genes, we further explored its effects on CRC. The IHC results from HPA also revealed that the expression level of CCR9 was significantly higher in colon cancer than in normal colon tissue (Fig. 12M). We then used HCT116 and SW480 cell lines with the highest expression levels for subsequent experiments. We designed a negative control group (NC) and two CCR9 knockdown groups (si-1 and si-2). We first verified the knockdown efficiency of si-1 and si-2 using qRT-PCR and Western blotting assays in HCT116 and SW480, respectively. The results exhibited that CCR9 was significantly down-regulated in both si-1 and si-2 in HCT116 (Fig. 12N,P) and SW480 (Fig. 12O,Q). Subsequently, we performed EdU and colony formation assays to examine proliferative ability. The results indicated that the proliferative abilities of si-1 and si-2 were significantly weaker than those of NC (Fig. 12R,S). The Transwell invasion assay was used to evaluate cell invasion capacity. As manifested in Fig. 12T, the number of invasive cells was significantly lower in si-1 and si-2 than in NC. The Transwell migration and wound healing assays were used to examine cell migration ability. As described in Fig. 12T, the number of migration cells was significantly lower in si-1 and si-2 than in NC. The 24-h scratch healing area of si-1 and si2 was significantly lower than that of NC (Fig. 12U). In conclusion, the aforementioned in vitro assays suggest that the high expression of CCR9 is closely related to poor prognosis in patients with CRC.

## Discussion

Developing gene multi-omics has enabled the construction of risk models based on multiple genes to predict the prognosis and immunotherapy response in colorectal adenocarcinoma patients. However, no study has used chemokines and chemokine receptors to establish a risk model in female advanced colorectal adenocarcinoma. Therefore, we first proposed the chemokine- and chemokine receptor-based signature to predict the prognosis of female advanced colorectal adenocarcinoma patients. Furthermore, the present study provides the first comprehensive understanding of the prognostic characteristics of chemokines and chemokine receptor families and their prognostic effect on immunotherapy in female advanced colorectal adenocarcinoma. First, we confirmed the robust prognostic significance of our risk model in female advanced colorectal adenocarcinoma patients from TCGA. Second, GO and KEGG enrichment analyses were performed to demonstrate that our risk model was significantly associated with immunity. Third, we explored the landscape of immune cell infiltration in female advanced colorectal adenocarcinoma patients; the results indicate that our risk model is closely correlated with T cells CD8 and neutrophils. Fourth, we examined the relationships between risk score and immune checkpoints and other immunotherapy-related biomarkers; the findings suggest that female advanced colorectal adenocarcinoma patients with lower risk scores are more sensitive to immunotherapy than those with higher risk scores. Fifth, we used ROC curves to examine the difference in prognostic power between our risk model and immune checkpoints and other immunotherapy-related biomarkers; the results confirmed that our risk model had better prediction performance than all immune checkpoints and immunotherapy-related biomarkers in female advanced colorectal adenocarcinoma patients. Sixth, we predicted chemotherapeutic drug sensitivity of our signature and established a nomogram using risk score, age, and stage. Finally, we verified the mRNA expression levels of signature genes and the adverse effects of CCR9 in CRC cell lines and tissues. The present study provides a comprehensive understanding of the role of a chemokine- and chemokine receptor-based signature in female advanced colorectal adenocarcinoma patients. The classification method based on our risk model would help clinicians better implement individualized clinical treatment for female patients with advanced colorectal adenocarcinoma.

Chemokines and chemokine receptors have been commonly reported to be involved in the occurrence and development of carcinomas and exist latent value in the immunotherapy of carcinomas^44,45^. In our study, six chemokines and chemokine receptors (CCL19, CCL22, CCR9, CXCR5, XCL1, and CX3CL1) were identified to establish the risk model. CCL19 attracts T cells and DCs through its receptor C-C chemokine receptor 7 (CCR7), thereby regulating cell innate and adaptive immunity^46–49^. Up-regulating the expression of CCL19 in tumors can inhibit its growth by effectively recruiting CCR7 + DC and IFN-γ + CD8 + T cells into tumor locations, which can be a powerful anti-tumor treatment combined with anti-PDL1^50^. CCL22 is overexpressed in colorectal adenocarcinomas^51^, which can facilitate Treg communication with DCs to control immunity by binding to C-C chemokine receptor 4 (CCR4) in lymph nodes^52^. CCR9 is the receptor of C-C motif chemokine ligand 25 (CCL25)^53^. Because of the expression of CCL25 in the intestinal tissues, lymphocyte homing to the tissues is the major function of the CCL25/CCR9 axis, which is of great significance in the immunological functions of the intestinal mucosa. This function results in intestinal metastasis of tumor cells with CCR9 expression^54^. CXCR5 is the receptor of C-X-C motif chemokine ligand 13 (CXCL13); CXCL13/CXCR5 signaling axis activity can accelerate the progression of colorectal carcinoma by activating the PI3K/AKT pathway^55^. The CXCL13/CXCR5 signaling axis can be used to predict the response of ICIs in colorectal carcinoma^56^. XCL1 is a kind of C-class chemokine, the receptor of X-C motif chemokine receptor 1 (XCR1). The XCL1/XCR1 axis plays a vital role in DC-mediated cytotoxic immune response^57^. CX3CL1 is a multifunctional inflammatory chemokine with a single receptor C-X3-C motif chemokine receptor 1 (CX3CR1), which can maintain the amount of effector memory cytotoxic T-cell populations in colorectal carcinoma^58^. The expression of CX3CL1 is negatively correlated with the prognosis of colorectal carcinoma patients^59^. In conclusion, the six chemokines and chemokine receptors enrolled in our risk model are closely associated with the prognosis and immune response of colorectal carcinoma.

We confirmed the robust prognostic significance of our risk model in female advanced colorectal adenocarcinoma patients through Kaplan–Meier survival and metastatic lymph node analyses firstly. Subsequently, to explore the potential mechanisms of the effect of our risk model on prognostic significance, we performed GO and KEGG enrichment analyses, which demonstrated that our risk model was closely associated with immune activity-related pathways in female advanced colorectal adenocarcinoma patients from TCGA. Therefore, we explored the landscape of immune cell infiltration. Tumor‐associated neutrophils (TANs) infiltrate the TME, which can regulate tumor progression^60^. According to the distinct effects of TANs on tumors, TANs are classified into anti-tumor (N1) and pro-tumor (N2) types^61^. N1 can inhibit tumor progression by increasing the cytotoxicity of TANs^62^, stimulating the adaptive immune system^63^, or reducing the anti-tumor immunosuppressive response^64^. N2 can promote tumor proliferation, metastasis, and invasion by releasing neutrophil extracellular traps (NETs)^65^ or inhibiting immune response^66^. After inhibition of TGF-β signaling or induction with IFN-β in tumors, TANs tend to differentiate towards the N1 phenotype^64,67^. When stimulating granulocyte colony-stimulating factor (G-CSF), TANs tend to differentiate towards the N2 phenotype^61^. In our study, the risk score was negatively correlated with neutrophils. Hence, the neutrophil in female advanced colorectal adenocarcinoma patients belonged to the N1 phenotype. Tumor-specific T CD8 cells are the core cellular components that exert anti-tumor effects in TME, which can dynamically respond to tumor antigen peptides presented by major histocompatibility complex II (MHC II) molecules in the presence of costimulatory or coinhibitory factors^68^. The elevated level of cytotoxic T CD8 cells is significantly associated with enhanced anti-tumor effects in colorectal carcinoma and other cancers^69^. Overexpression of CCXR2 and S1PR4 can inhibit infiltration of T cells CD8 in colorectal carcinoma^32,33^. In our study, the risk score was positively related to T CD8 cells. Consequently, the infiltration level of T CD8 cells could be reversed by the overexpression of CCXR2 and S1PR4 in female advanced colorectal adenocarcinoma patients. Of course, there must be other factors that can affect the immune infiltration level of T CD8 cells, which requires further study.

In our study, the primary objective was to explore relationships between our risk model and immune checkpoints and other immunotherapy-related biomarkers, which could prove the predictive value of our risk model for immunotherapy response in female advanced colorectal adenocarcinoma patients. PD1, CTLA4, PDL1, PDL2, LAG3, and TIM3 are commonly immune checkpoints, proven to be immunotherapy biomarkers^70–72^. ICIs have made an indelible mark in the field of advanced tumor immunotherapy. Starting with the approval of anti-CTLA4 for advanced melanoma in 2011, anti-PD-1 and anti-PDL1 also gained approval from the United States Food and Drug Administration to treat abundant tumor types, indicating unprecedented survival extension of patients with advanced carcinoma^73^. Despite the success of ICI treatment, resistance to these ICIs restricts many patients with advanced carcinoma who are unable to benefit from immunotherapy. Thus, it is necessary to understand immunotherapy response to ICIs better. In our study, risk score was significantly negatively correlated with CTLA4, PDL1, and PDL2, indicating that female advanced colorectal adenocarcinoma patients with lower risk scores are more likely to benefit from immunotherapy of anti-CTLA4, anti-PDL1, and anti-PDL2. TMB is the total number of base mutations per 1,000,000 somatic cells in tumor^74^. Despite prior researchers having proved that higher TMB is closely related to better prognosis in many kinds of tumors^75^, its prognostic significance in colorectal carcinoma remains unclear. The higher TMB was significantly related to the better efficacy of immunotherapy in colorectal carcinoma^37^. Meanwhile, a study reported that adjuvant chemotherapy for colorectal carcinoma patients with TMB > 8 had a higher survival rate than those with TMB < 8^40^. In our study, although there was no significant correlation between risk score and TMB, the proportion of female advanced colorectal adenocarcinoma patients with TMB > 8 in the low-risk group was higher than that in the high-risk group. The results suggest that female advanced colorectal adenocarcinoma patients in the low-risk group may be more likely to benefit from chemotherapy than those in the high-risk group. A microsatellite is a simple, repetitive, and highly mutable sequence in the genome. Microsatellite gain or loss mutations occur during the DNA replication process, referred to as MSI. MSI is primarily caused by mutations in mismatch repair (MMR) genes (including PMS2, MSH6, MSH2, and MLH) or abnormal expression of MMR deficient (d-MMR)^3^. Because of the lack of intratumor heterogeneity, colorectal carcinoma patients with high MSI have better efficacy, lower drug resistance, and lower failure rate of ICI treatment compared with those with low MSI^76,77^. Meanwhile, several studies have proved that colorectal carcinoma patients with high MSI have better chemotherapy efficacy than those with low MSI^40,43,78^. In our study, the proportion of female advanced colorectal adenocarcinoma patients with high TMB in the low-risk group was significantly higher than that in the high-risk group, indicating that female advanced colorectal adenocarcinoma patients in the low-risk group are more likely to benefit from immunotherapy and chemotherapy than those in the high-risk group. TIDE is a more accurate biomarker than other immunotherapy-related biomarkers. T-cell dysfunction in tumors with high infiltration of cytotoxic T lymphocytes (CTL), whereas T-cell exclusion was presented in tumors with low T-cell invasion^26^. In our study, risk score was negatively related to T-cell dysfunction score, whereas risk score was positively correlated with T-cell exclusion score. These findings suggest again that female advanced colorectal adenocarcinoma patients with lower risk scores are more likely to benefit from immunotherapy. compared with those with higher risk scores Based on the aforementioned results, our risk model could also serve as a robust biomarker for immunotherapy and chemotherapy response in female advanced colorectal adenocarcinoma patients.

Finally, to assess the superiority of the risk model in female advanced colorectal adenocarcinoma patients, we performed ROC curves to compare the prognostic power of the chemokine- and chemokine receptor-based signature with ICIs and other biomarkers. The results demonstrated that the prognostic power of our risk model was significantly higher than ICIs and other biomarkers in female advanced colorectal adenocarcinoma patients.

Although the chemokine- and chemokine receptor-based signature can serve as a robust biomarker for immunotherapy and chemotherapy response in female advanced colorectal adenocarcinoma patients, our study has some limitations. First, all samples from four independent cohorts were retrospective data, and a prospective study of the risk model will be necessary. Second, validation was only performed using 32 pairs of tissue specimens and two CRC cell lines, which might not encompass the heterogeneity of female advanced colorectal adenocarcinoma fully. Third, our study only demonstrated the relationship between signature and immune checkpoints, however, we didn’t explore the relationship between the signature and other potential influencing factors (such as patient lifestyle or other genetic markers). Finally, the ability to predict the immunotherapy and chemotherapy response was assessed indirectly; a more direct study will be required to validate our results.

In conclusion, we first constructed a signature based on chemokines and chemokine receptors in female advanced colorectal adenocarcinoma patients, which could be used to predict the prognosis of female patients with advanced colorectal adenocarcinoma. Meanwhile, the landscape of immune infiltration and immune response were described. Finally, the signature could also serve as a biomarker for predicting immunotherapy and chemotherapy response in female advanced colorectal adenocarcinoma patients, which will provide significant guidance for clinicians to achieve individualized immunotherapy and chemotherapy for female patients with advanced colorectal adenocarcinoma.

## Conclusions

Our study was the first to construct a robust prognostic chemokine- and chemokine receptor-based signature, which could serve as a new guideline for immunotherapy and chemotherapy response to provide individualized treatment strategy for female patients with advanced colorectal adenocarcinoma.

### Supplementary Information

Supplementary Figure S1.

Supplementary Figure S2.

Supplementary Figure S3.

Supplementary Figure S4.

Supplementary Figure S5.

Supplementary Figure S6.

Supplementary Table 1.

Supplementary Table 2.

Supplementary Table 3.

Supplementary Table 4.

Supplementary Table 5.

## Data Availability

Publicly available datasets were analyzed in this study. These data can be found here: GSE17536 (https://www.ncbi.nlm.nih.gov/geo/query/acc.cgi?acc=GSE17536), GSE17537 (https://www.ncbi.nlm.nih.gov/geo/query/acc.cgi?acc=GSE17537), GSE39582 (https://www.ncbi.nlm.nih.gov/geo/query/acc.cgi?acc=GSE39582), and TCGA (https://portal.gdc.cancer.gov/).

## Abbreviations

DEGs: Differentially expressed genes
GEO: Gene Expression Omnibus
GO: Gene Ontology
GSEA: Gene set enrichment analysis
CRC: Colorectal cancer
HPA: Human Protein Atlas
KEGG: Kyoto Encyclopedia of Genes and Genomes
PCA: Principal component analysis
qRT-PCR: Quantitative real-time PCR
TCGA: The Cancer Genome Atlas
TMB: Tumor mutation burden
TME: Tumor microenvironment
