# Supplementary material for: Chemokine- and chemokine receptor-based signature predicts immunotherapy response in female colorectal adenocarcinoma patients
Source: Sci Rep. 2023 Dec 4;13:21358. doi: 10.1038/s41598-023-48623-2 (PMC10695967; doi:10.1038/s41598-023-48623-2)
Supplement: Supplementary file 2 — Supplementary Figure S2. [file 41598_2023_48623_MOESM2_ESM.pdf]

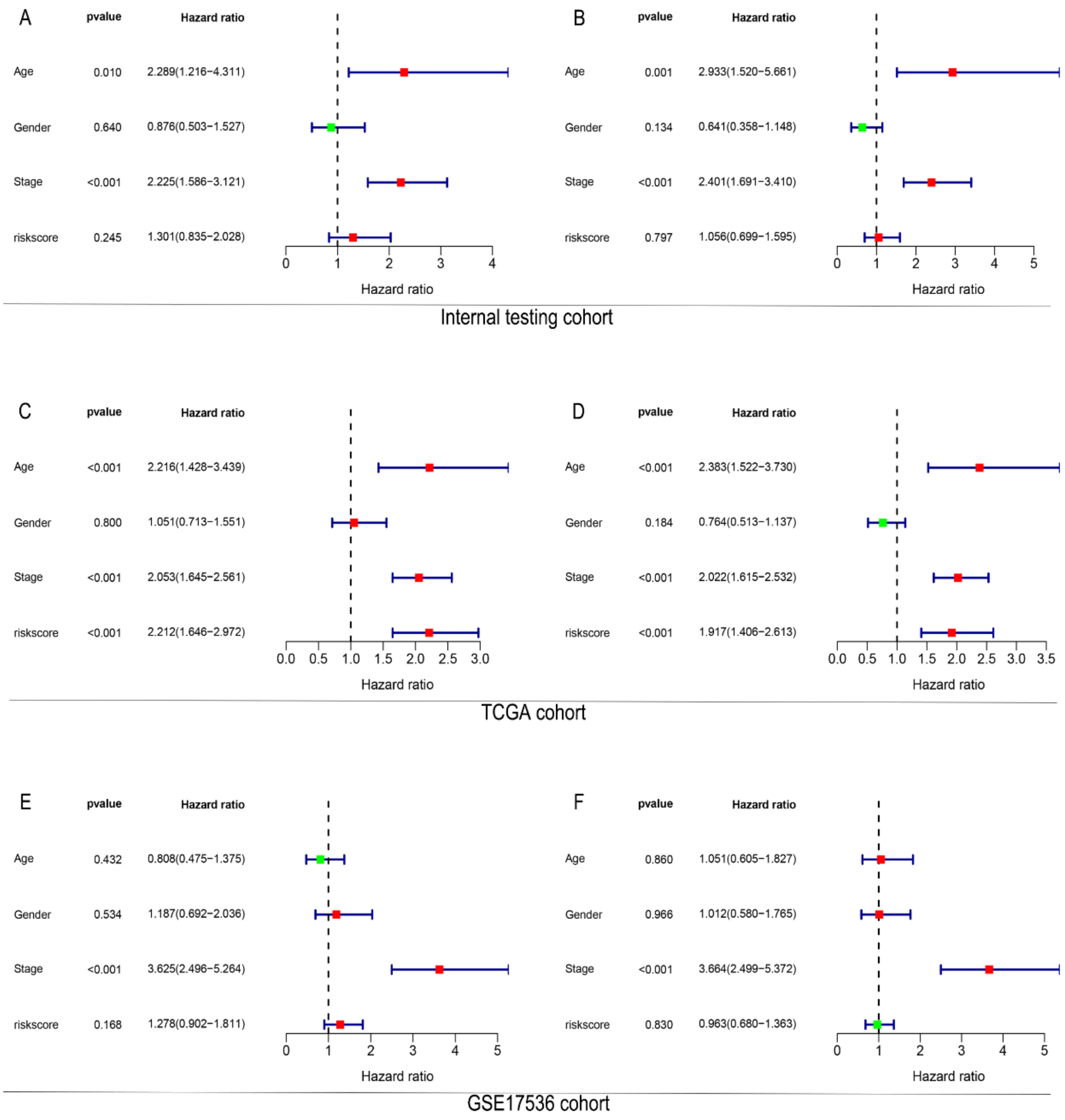

Figure S2. Univariate and multiple Cox regression analyses in Internal testing cohort, TCGA cohort, and GSE17536 cohort. **(A)** Univariate Cox regression analysis in Internal testing cohort. **(B)** Multiple Cox regression analysis in Internal testing cohort. **(C)** Univariate Cox regression analysis in TCGA cohort. **(D)** Multiple Cox regression analysis in TCGA cohort. **(E)** Univariate Cox regression analysis in GSE17536 cohort. **(F)** Multiple Cox regression analysis in GSE17536 cohort.
