# Supplementary material for: Chemokine- and chemokine receptor-based signature predicts immunotherapy response in female colorectal adenocarcinoma patients
Source: Sci Rep. 2023 Dec 4;13:21358. doi: 10.1038/s41598-023-48623-2 (PMC10695967; doi:10.1038/s41598-023-48623-2)
Supplement: Supplementary file 3 — Supplementary Figure S3. [file 41598_2023_48623_MOESM3_ESM.pdf]

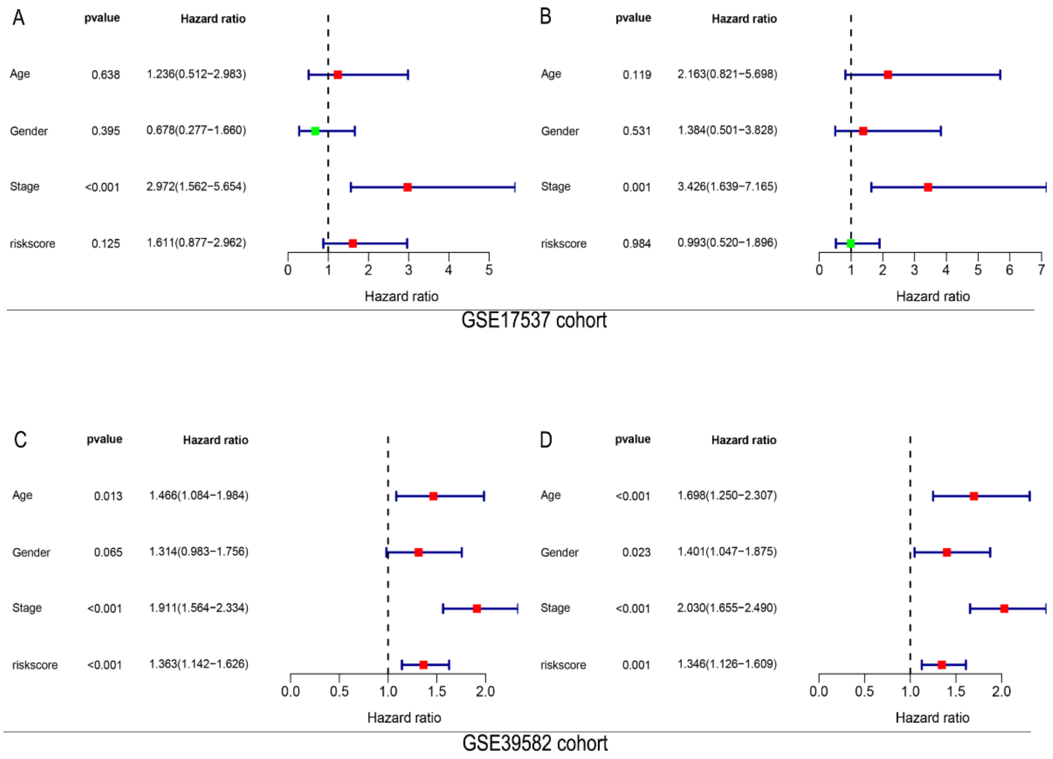

Figure S3. Univariate and multiple Cox regression analyses in GSE17537 cohort and GSE39582 cohort. **(A)** Univariate Cox regression analysis in GSE17537 cohort. **(B)** Multiple Cox regression analysis in GSE17537 cohort. **(C)** Univariate Cox regression analysis in GSE39582 cohort. **(D)** Multiple Cox regression analysis in GSE39582 cohort.
