# Supplementary figures and images for: Chemokine- and chemokine receptor-based signature predicts immunotherapy response in female colorectal adenocarcinoma patients
Source: Sci Rep. 2023 Dec 4;13:21358. doi: 10.1038/s41598-023-48623-2 (PMC10695967; doi:10.1038/s41598-023-48623-2)

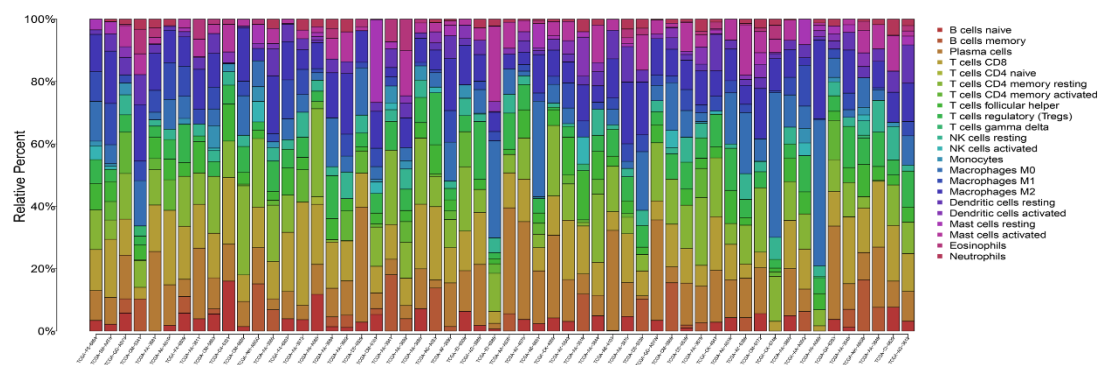

**Figure S4.** The percentage of 22 immune cells infiltration in each TCGA sample.

Supplement: Supplementary file 4 — Supplementary Figure S4. [file 41598_2023_48623_MOESM4_ESM.pdf]
