# Supplementary material for: Chemokine- and chemokine receptor-based signature predicts immunotherapy response in female colorectal adenocarcinoma patients
Source: Sci Rep. 2023 Dec 4;13:21358. doi: 10.1038/s41598-023-48623-2 (PMC10695967; doi:10.1038/s41598-023-48623-2)
Supplement: Supplementary file 5 — Supplementary Figure S5. [file 41598_2023_48623_MOESM5_ESM.pdf]

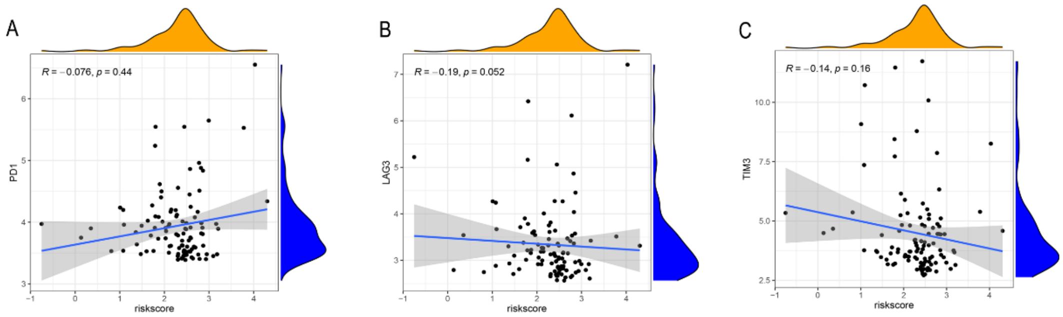

Figure 5. Correlations between risk score and immune checkpoints in Female + Stage III-IV cohort. Correlations between risk score and PD1 **(A),** LAG3 **(B)**, and TIM3 **(C)** in Female + Stage III-IV cohort.
