# Supplementary material for: Chemokine- and chemokine receptor-based signature predicts immunotherapy response in female colorectal adenocarcinoma patients
Source: Sci Rep. 2023 Dec 4;13:21358. doi: 10.1038/s41598-023-48623-2 (PMC10695967; doi:10.1038/s41598-023-48623-2)
Supplement: Supplementary file 6 — Supplementary Figure S6. [file 41598_2023_48623_MOESM6_ESM.pdf]

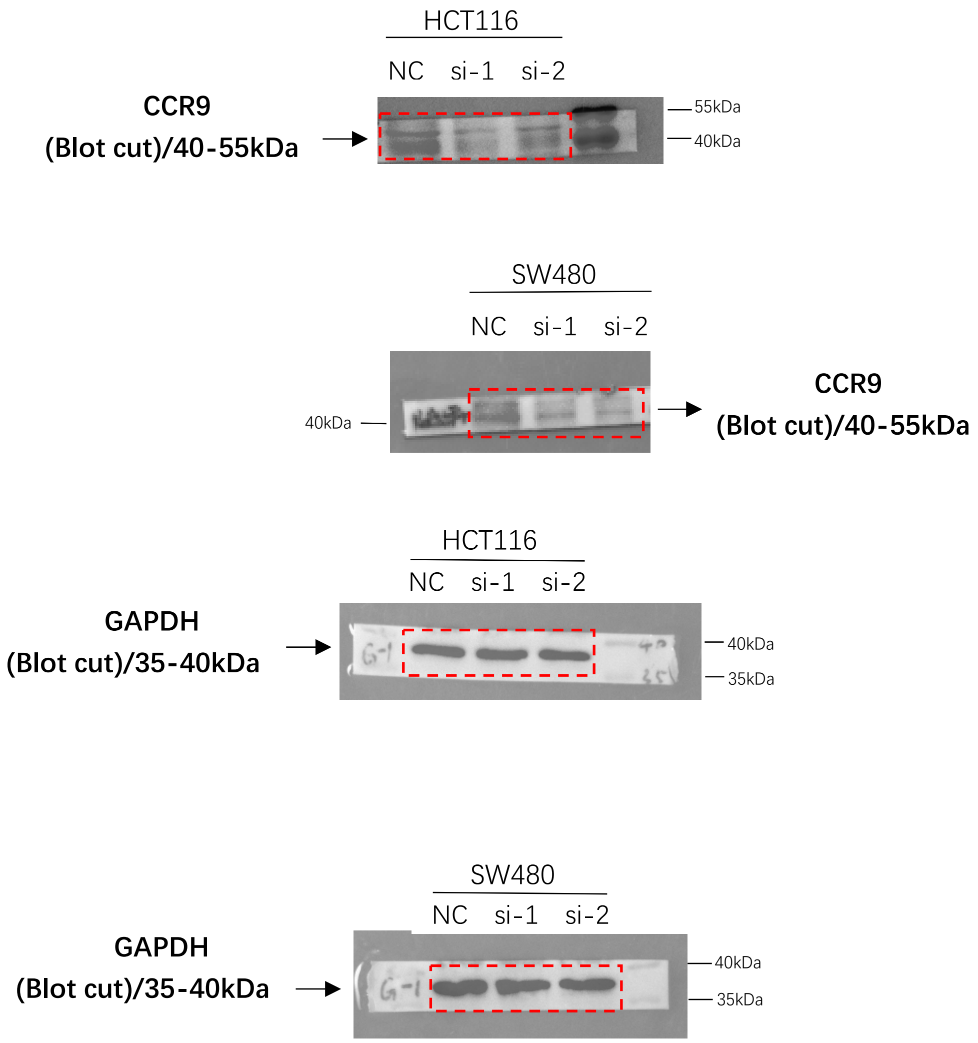

FigureS6. Full-length western blot images of Figure 12K-L. HCT116 and SW480 cells were transfected with CCR9 siRNA. And western blotting was used to determine the expression level of CCR9. GAPDH was used as a loading control.
