# Supplementary material for: Chemokine- and chemokine receptor-based signature predicts immunotherapy response in female colorectal adenocarcinoma patients
Source: Sci Rep. 2023 Dec 4;13:21358. doi: 10.1038/s41598-023-48623-2 (PMC10695967; doi:10.1038/s41598-023-48623-2)
Supplement: Supplementary file 1 — Supplementary Figure S1. [file 41598_2023_48623_MOESM1_ESM.pdf]

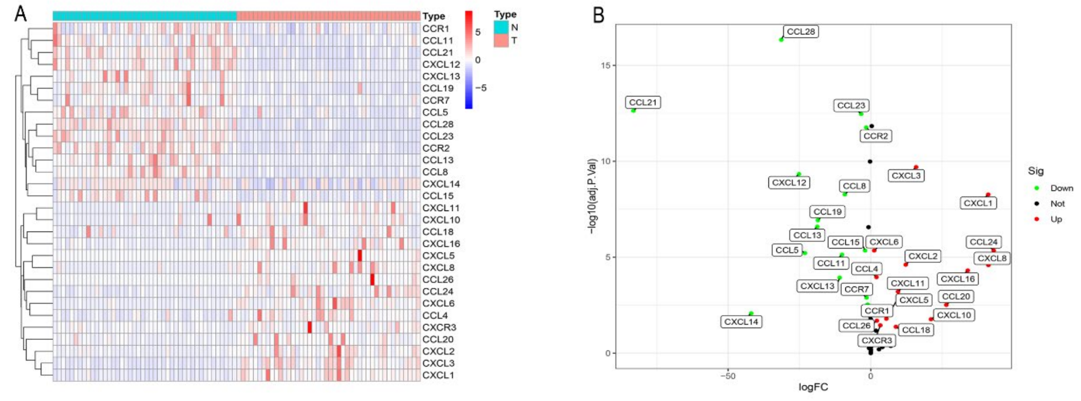

Figure S1. Heatmap **(A)** and volcano plot **(B)** presented the DEGs between 44 pairs of colorectal adenocarcinoma tissues and adjacent normal tissues from TCGA cohort.
